# Single-cell integrative analysis reveals consensus cancer cell states and clinical relevance in breast cancer

**DOI:** 10.1038/s41597-024-03127-0

**Published:** 2024-03-12

**Authors:** Lin Pang, Fengyu Xiang, Huan Yang, Xinyue Shen, Ming Fang, Ran Li, Yongjin Long, Jiali Li, Yonghuan Yu, Bo Pang

**Affiliations:** College of Bioinformatics Science and Technology, Harbin Medical University, Harbin, 150081 China

**Keywords:** Breast cancer, Tumour heterogeneity

## Abstract

High heterogeneity and complex interactions of malignant cells in breast cancer has been recognized as a driver of cancer progression and therapeutic failure. However, complete understanding of common cancer cell states and their underlying driver factors remain scarce and challenging. Here, we revealed seven consensus cancer cell states recurring cross patients by integrative analysis of single-cell RNA sequencing data of breast cancer. The distinct biological functions, the subtype-specific distribution, the potential cells of origin and the interrelation of consensus cancer cell states were systematically elucidated and validated in multiple independent datasets. We further uncovered the internal regulons and external cell components in tumor microenvironments, which contribute to the consensus cancer cell states. Using the state-specific signature, we also inferred the abundance of cells with each consensus cancer cell state by deconvolution of large breast cancer RNA-seq cohorts, revealing the association of immune-related state with better survival. Our study provides new insights for the cancer cell state composition and potential therapeutic strategies of breast cancer.

## Introduction

Breast cancer is the most commonly diagnosed cancer^1^ and a leading cause of cancer-related mortality in women^2^. The efficacies of therapies targeting key genes, like the estrogen receptor (ER), human epithelial growth factor receptor 2 (HER2/ERBB2), the mammalian target of rapamycin (mTOR) and the epidermal growth factor receptor (EGFR), were influenced by the heterogeneous cancer cells and tumor-associated cells which were characterized by diverse transcriptional or mutational states^3–5^. Our lack of understanding of the tumor heterogeneity is a major obstacle for implementation of precision medicine^6^.

Breast cancer is a highly heterogeneous disease, which is clinically stratified based on the expression of ER, progesterone receptor (PR), and HER2 into three broad clinical groups that correlate with prognosis and treatment strategies: ER+, HER2+, and triple-negative breast cancer (TNBC)^7^. Alternative classification based on gene expression have been also proposed^8–10^. Although these stratifications bring improvement of therapy success and survival rates, the tumor cell heterogeneity exists and patient responses vary within each subtype^6^, demanding a deeper understanding of cellular compositions and their associations with clinical phenotype.

Since breast cancer intrinsic subtypes showed prominent resemblance to distinct breast epithelial cells, which may reflect cells of origin across subtype^2^, many studies focused on the mammary cell hierarchy and its relation with breast cancer^11,12^. For example, Bhat-Nakshatri *et al*. performed single-cell RNA sequencing (scRNA-seq) of breast biopsies of healthy women and identified three main cell groups including basal/stem, luminal progenitor and luminal mature cells. They further obtained 23 clusters and mapped them to bulk transcriptome of breast cancer patients for each molecular subtype^13^. Recently, the Human Breast Cell Atlas (HBCA) was constructed to create a comprehensive atlas of cell types and cell states in normal breast tissues, providing valuable reference for studying the mechanism of tumorigenesis and progression of breast cancer^14^. Moreover, single-cell analyses of tumor tissues also provided insights into the cellular architecture of breast cancer and refinement of molecular signatures correlated with malignant transformation and clinical subtype^7,15,16^. However, the incomplete consistency of results among those studies and their limited clinical value declare the large insufficiency of our understanding of the cellular heterogeneity. Further systematic evaluation of tumor cell state compositions, their functions and origins, as well as their contributions to breast cancer subtype and clinical outcomes is required.

Single-cell RNA sequencing (scRNA-seq) is a revolutionary technique that enables an unbiased dissection of the spectrum of heterogeneous subpopulations and the transcriptional states of single cells within breast cancer tissues. Here we elucidated the consensus cancer cell states across breast cancer patients by integrative analysis of scRNA-seq data. Cancer cells with each state had distinct functions and subtype-specific distribution, which were validated in another three sets of scRNA-seq data of breast cancer. We dissected the intrinsic factors and those in the microenvironment that contributed to diverse cancer cell states, the relations of each state with clinical phenotype and outcomes. Our study provides new insights for the consensus cellular state composition of breast cancer and potential therapeutic strategies.

## Results

### The relatively minor consistence between the genomic and transcriptomic heterogeneity

**Fig. 1:**
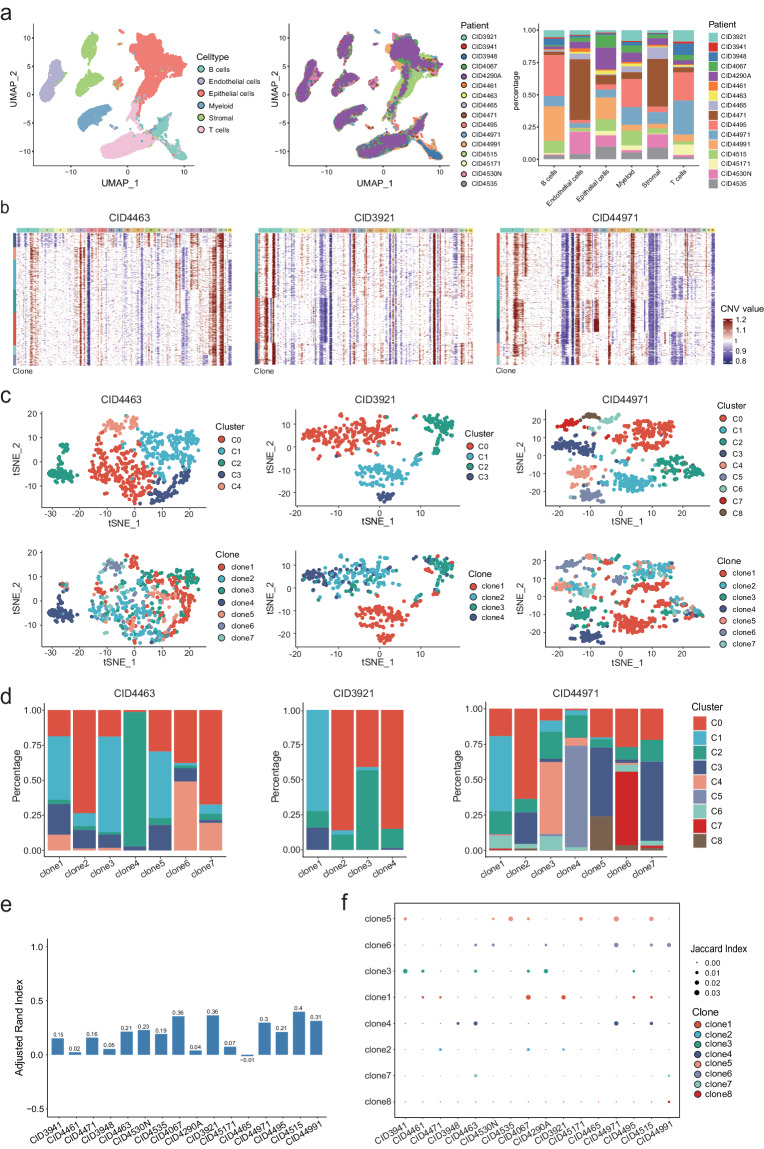
The association between genomic and transcriptomic heterogeneity in breast cancer. (**a**) UMAP plots of major cell types (left) and patients (middle), and distribution of cell counts across major cell types, colored by patients (right). (**b**) In patients CID4463, CID3921, and CID44971, inferCNV heatmaps of all malignant cells along with their respective subclones, identified based on the inferred copy number alteration of all patients. Each row represents a cell and column represents a gene. (**c**) TSNE visualization displaying transcriptional clusters (top) and subclones (bottom). (**d**) Relative proportions of transcriptional clusters within each subclone. (**e,f**) Assessment of ARI (**e**) and Jaccard index (**f**) measuring the concordance between genomic and transcriptomic heterogeneity across 16 patients.

To decipher the tumor cellular composition of breast cancer cells, we downloaded the scRNA-seq data of 26 primary breast cancer patients, which was published by Wu *et al*.^17^. After quality control filtering, we performed unsupervised clustering analyses, which captured six major cell types: epithelial cells, B cells, T cells, stromal, endothelial cells, myeloid, each defined by singleR^18^ and canonical markers (see Methods). We further identified malignant cells using large-scale chromosomal copy number variation (CNV) inferred by inferCNV^19^ (see Methods). After removing patients without malignant cells, a total of 65,437 cells with 16,681 detected genes were retained from 16 patients (Supplementary Table 1), and each cell type contained cells from the majority of patients (Fig. 1a).

To determine the contribution of genomic changes to transcriptomic heterogeneity, we first inferred the tumor subclonal architecture in each patient based on the CNV profiles (Fig. 1b and Supplementary Fig. 1, see Methods). The numbers of subclones were from 3 to 8 with a median of 5 across patients, suggesting high genomic heterogeneity. We then re-clustered malignant cells in each patient and aimed to explore whether a subclone tended to enrich specific transcriptional cluster (Fig. 1c and Supplementary Fig. 2). We did not observe the correspondence between them, as most subclones contained cells within at least two transcriptional clusters (Fig. 1d and Supplementary Fig. 3), although a specific subclone may be predominantly enriched for one transcriptional cluster, such as clone4 in CID4463. To confirm this, the adjusted Rand index^20^ (ARI) was adopted as measure of agreement between the genomic and transcriptional heterogeneity. Relatively low ARIs were observed for all patients (Fig. 1e). Moreover, for each subclone, we used the Jaccard similarity coefficient to measure the overlaps between the differential expression genes and subclone-specific CNV genes (Fig. 1f, see Methods). The results showed very low Jaccard similarity coefficients for all subclones across patients, suggesting partial contributions of genomic changes to the transcriptional heterogeneity of breast cancer cells.

### Identifying consensus states of breast cancer cells

**Fig. 2:**
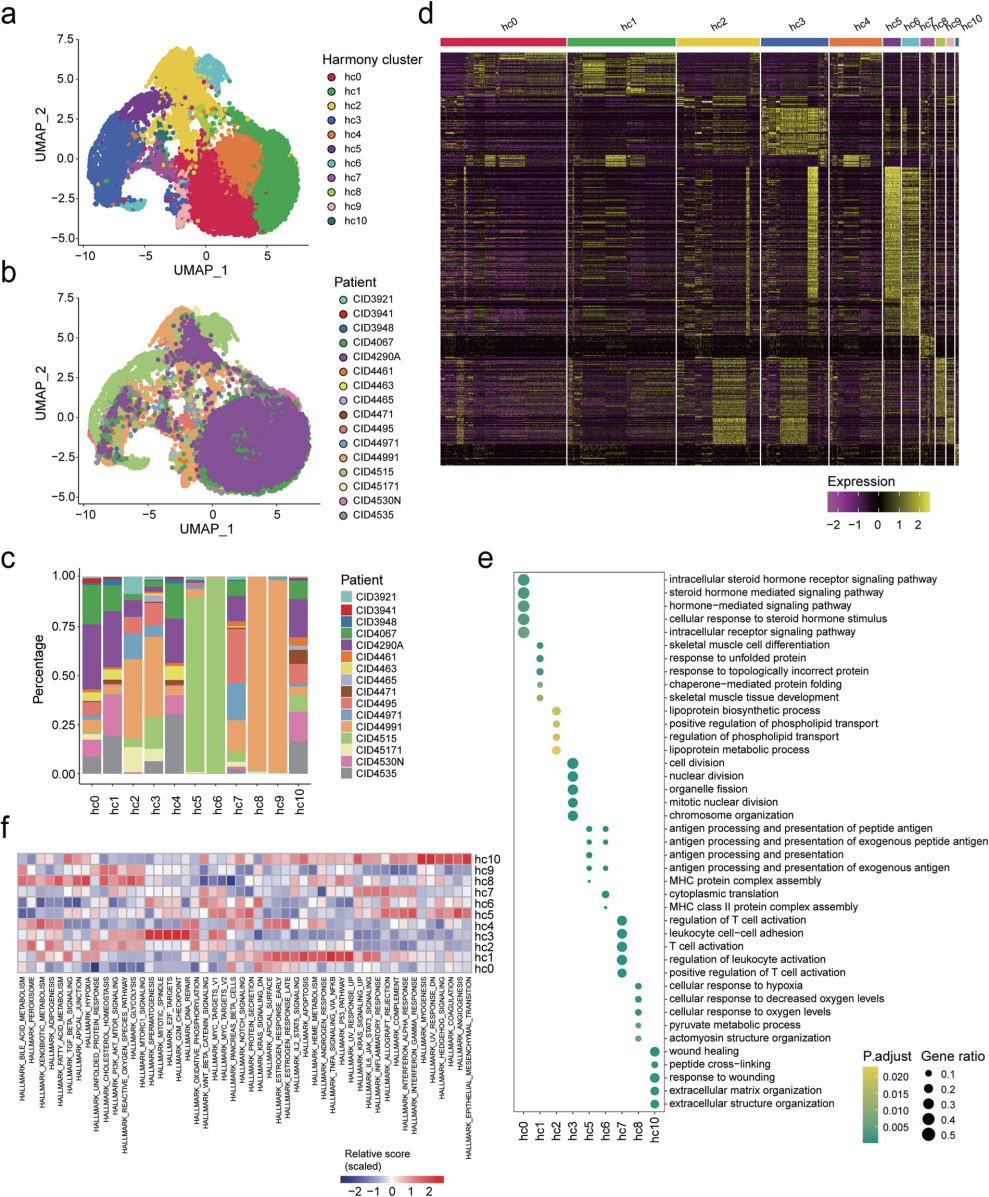
Consensus cell state map of breast cancer cells. (**a,b**) UMAP visualization of 21,521 malignant cells, colored by harmony cluster (**a**) and patients (**b**). (**c**) The percentages of cells from all patients in each harmony cluster. (**d**) The heatmap displaying scaled expression values of discriminative genes per cell cluster as defined in the panel. (**e**) Bubble diagram showing the representatives of enriched GO terms for each cluster. (**f**) Heatmap showing the relative activity scores of cancer hallmark gene sets (n = 50) in each harmony cluster.

Next, we focused on the transcriptomic heterogeneity of breast cancer cells. In order to determine the cancer cell states that are generalizable across patients, we integrated the expression profiles of malignant cells from all the 16 patients using Harmony^21^ (see Methods). This analysis generated 11 clusters of cells (hc0-hc10, Fig. 2a,b). Each cluster contained cells from most patients with different proportions (Fig. 2c), except for hc5, hc6, hc8 and hc9, where hc5 and hc6 were mostly composted of cells from CID4515, while hc8 and hc9 were mostly composted of cells from CID44991. To learn more about the biological functions underlying these cancer cell clusters, we identified their significantly upregulated genes as signatures (Fig. 2d and Supplementary Table 2) and performed functional enrichment analysis. The results demonstrated quite distinct biological processes involved by each cluster, except for hc4 and hc9 (Fig. 2e). PIP, SCGB2A2, ESR1 were enriched in hc0 (Supplementary Fig. 4a, Supplementary Table 2), which was associated with hormone-mediated signaling pathway and response. ARG2, ARG3, IFI6 and IFI27 were enriched in hc1 and hc4, which was involved in muscle cell differentiation and protein folding. Hc2 highly expressed KRT7, KRT81 and ERBB2, which participated in lipoprotein metabolic process. Hc3 displayed high cell proliferation activity as it was almost exclusively enriched for cell cycle-related genes, such as CDC20, CDK1 and MKI67. Immune-related biological processes were enriched in hc5, hc6 and hc7, where both hc5 and hc6 were involved in antigen processing and presentation, while hc7 was additionally enriched for the regulation of immune cells activation. Hc8 showed the activation of metabolic processes, such as cellular response to hypoxia and glycolysis. Hc10 were enriched for mesenchymal-related markers, such as VIM, COL1A1 and ACTA2, which was involved in wound healing, extracellular matrix organization and angiogenesis. Cancer hallmarks analysis by gene set variation analysis (GSVA) validated above function annotation, as the meiosis, checkpoint and DNA repair pathways were enriched in cells with state hc3 while the EMT, angiogenesis and coagulation pathways were enriched in cells with state hc10 (Fig. 2f). Given that hc5, hc6, hc8 and hc9 were individual-specific (Supplementary Fig. 4b), we considered that hc0-hc4, hc7 and hc10 respectively represent seven consensus cancer cell states (CCSs) with distinct functions in breast cancer.

### Systemic characterization of consensus cell states and their intrinsic driver factors

**Fig. 3:**
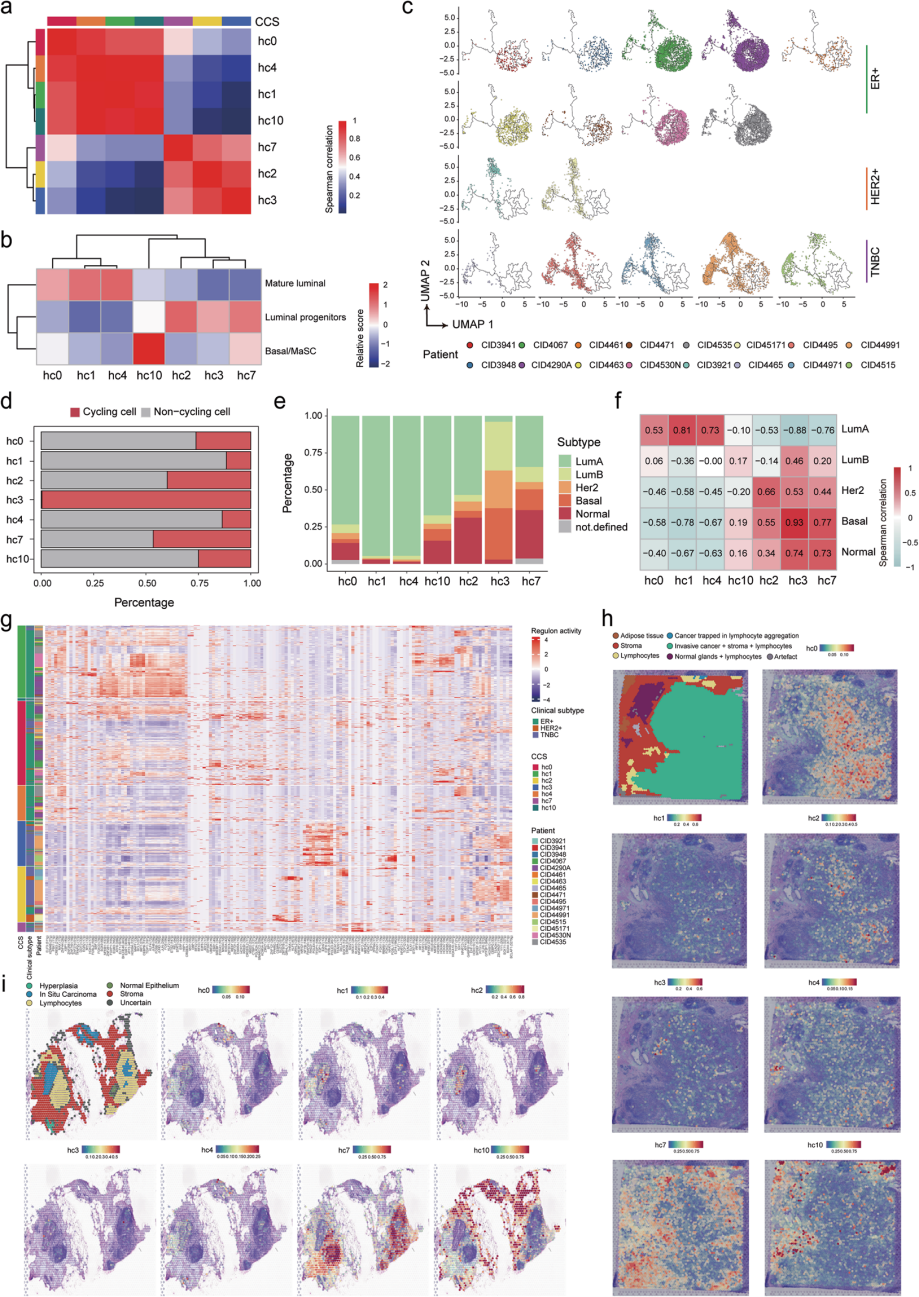
Dissection of consensus cell states (CCSs) and their intrinsic driver factors. (**a,b**) Heatmap illustrating spearman correlation coefficients (computed using DEGs) between CCSs (**a**) and the lineage identity of cells, including mature luminal, luminal progenitors, and Basal/MaSC (**b**). (**c**) Pseudo-time trajectory of malignant cells in each tumor, with patients grouped by their clinical subtypes. (**d**) Proportion of cycling (cells in G2M or S phase) and non-cycling cells across the consensus cell states. (**e**) Percentage distribution of molecular subtypes (LumA, LumB, Her2, Basal, and Normal) assigned to each of the seven CCSs. (**f**) Spearman correlation coefficient indicating the relationship between the seven CCSs and each molecular subtype. (**g**) Heatmap depicting the relative activities of regulons in cells of each CCS, accompanied by patient and clinical subtype information. (**h,i**) Pathology annotations and localization of CCSs based on CARD deconvolution: sample 1160920 F from ST_Wu_2021 (**h**), and sample M11 from ST_Angele_2023 (**i**). The proportion of CCSs for each spot was calculated using CARD. Red indicates a higher proportion, and blue indicates a lower proportion.

To understand how those consensus cell states are related to each other, we calculated the spearman correlation in a pairwise manner, which showed two highly correlated groups (Fig. 3a), with one containing hc0, hc1, hc4 and hc10 (denoted G1), and the other containing hc2, hc3 and hc7 (denoted G2). Given the dominant role of cell-of-origin patterns in cancers^22^, we determined the lineage identity for each CCS by computing expression scores using bulk RNA-seq signatures of major epithelial cell types in the breast^23,24^. States hc0, hc1 and hc4 resemble “mature luminal” cells, states hc2, hc3 and hc7 correspond closely to “luminal progenitors” while hc10 matched with cell population designated as “Basal/MaSC” (Fig. 3b). To validate this, lineage identity was also determined using scRNA-seq signatures^14^. Similar results were observed that hc0, hc1 and hc4 correspond to luminal cells designated as “LumHR” and “LumSec”, hc3, hc7 and hc10 resemble “basal” cells, while hc2 showed high scores of both “LumSec” and “basal” (Supplementary Fig. 5a). Since studies have reported that basal cells contain a subset of mammary stem cells (MaSCs)^25^, we evaluated the differentiation potential of each cell using CytoTRACE^26^ and SCENT^27^. As expected, hc2, hc3, hc7 and hc10 exhibit higher scores than the others, representing higher stemness (Supplementary Fig. 5b,c). And when we reconstructed differentiation trajectory using all the cells of seven CCSs by Monocle^28^ (see Methods), we found that cells from HER2+ /TNBC patients distributed in one branch, whereas those from ER+ patients located in the other two different branches (Fig. 3c). These results were accordant with that G1 was enriched in ER+ patients while G2 was enriched in HER2+ /TNBC patients (Supplementary Fig. 4b), supporting the report that HER2+ breast cancers may originate from luminal progenitors and mature luminal cells, while luminal-A/B breast cancers likely originate from mature luminal cells^29^.

We next examined the tumor cell proliferative property and assessed the proliferation fractions of cells. Each individual cell was assigned a cell-cycle state based on its expression score of cell-cycle-related signature (see Methods). The results suggested that hc3 had the highest proliferation rate, consistent with its function annotation, followed by hc7 and hc2 (Fig. 3d). Considering that the intrinsic subtypes of breast cancer also likely reflect distinct features and origins of cells, we aimed to explore the linkage between CCS and molecular subtype. PAM50 assignment of each cell was performed using genefu^30^, showing higher fractions of LumA and LumB in G1 and higher fractions of Basal or Normal in G2 (Fig. 3e). Moreover, we calculated the spearman correlation coefficient between the proportion of each molecular subtype and that of each CCS across patients. This analysis suggested that G1 was mainly associated with LumA/B while G2 was related with Her2, Basal and Normal (Fig. 3f). Notably, hc10 showed partial association with Basal, suggesting a mixed state to some extent, while hc3 showed extensive associations with each subtype except for LumA, suggesting the wide distribution of cycling cells.

To reveal the potential molecular basis driving the consensus cancer cell states, we utilized single-cell regulatory network inference and clustering (SCENIC)^31^ to identify the underlying regulatory network (see Methods). Generally, hc0, hc1, hc4 and hc10, referring to most cells from ER+ patients, showed higher activities of ELF3, EGR2/3, SOX9, JUNB and FOSB, while hc2, hc3 and hc10, referring to most cells from HER2+ /TNBC patients, showed higher activities of TEAD4, YY1, SOX11, SOX4 and BCLAF1 (Fig. 3g). Moreover, each CCS was also driven by relevant TFs (Supplementary Fig. 5d and Supplementary Table 3), including EP300, SP1, and HCFC1 for hc0; MAFF, BCL3, and MYC for hc1; THRA, CEBPA, and GRHL2 for hc2; EZH2, E2F8, and NFYB for hc3; SIX1, ZNF44, and TCF7 for hc4; NFATC2, RUNX3, and NR3C1 for hc7; and TEAD1, MEF2A for hc10. Notably, EP300 is one of the critical components of the core ER signaling complex, which could facilitate disease progression by potentiating ER signaling in breast cancer^32^, showing high activity in hc0. RUNX3 was enriched in hc7, which is a critical transcription factor playing essential roles in development and carcinogenesis and has been frequently reported to be a tumor suppressor in breast cancer through diverse mechanisms^33^. These results demonstrated that cells with each CCS contributed to the carcinogenesis and progression through distinct regulators-mediated signaling pathways in breast cancer.

To reveal the distribution of CCSs, we accessed public spatial transcriptomics (ST) data of 12 breast cancer samples from three datasets, including ST_Wu_2021^34^, ST_Angele_2023^35^, and ST_Amanda_2023^36^ (see Methods). After data processing, conditional autoregressive-based deconvolution (CARD)^37^ was applied to calculate the proportion of CCSs at each spatial location (see Methods). The results showed that seven CCSs exhibited consistent regional specificity in three datasets (Fig. 3h,i, and Supplementary Fig. 6). For example, hc7 (immune state) tended to be enriched in the regions with lymphocyte infiltration and inflammation, proximity to the peripheral zone of the tumor. However, hc10 was enriched in the stroma regions around tumor margin, which was consistent with the EMT-associated functional state. Other CCSs (hc0, hc1, hc2, hc3, and hc4) were mainly inside the tumor region. As a validation, multimodal intersection analysis (MIA)^38^ was also used to generate the corresponding relationship between the CCSs and ST tissue-specific locations (see Methods), and we observed similar results (Supplementary Fig. 7).

### Validation of consensus cell states in extra scRNA-seq datasets of breast cancer

To determine whether the consensus cell states and their relations with subtypes can be reproduced, we obtained another three scRNA-seq datasets. The processing of these validation data was similar with Wu_2021 (see Methods).

**Fig. 4:**
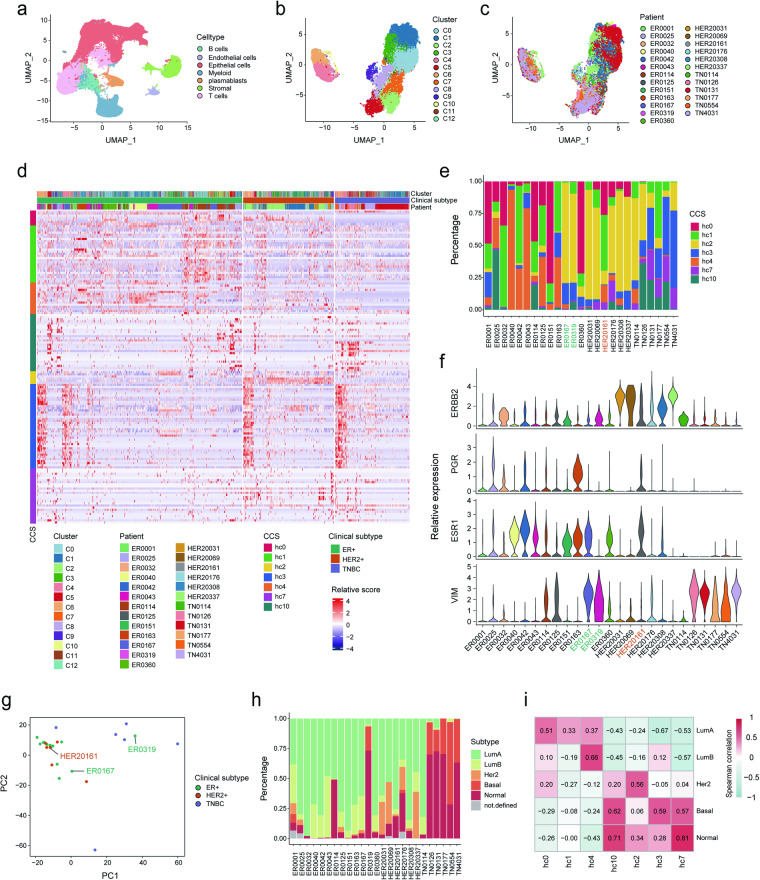
The consistency verification of CCSs, clinical subtypes, and molecular subtypes in the Bhupinder_2021 dataset. (**a**) UMAP visualization of all cells in the Bhupinder_2021 cohort. Clusters were annotated based on predicted cell types using canonical markers. (**b,c**) Clusters of all malignant cells from patients with at least 10 malignant cells, colored by clusters (**b**) and patients (**c**). (**d**) The expression levels of signature genes of CCSs within cancer cells of each clinical subtype. (**e**) Percentage distribution of CCSs assigned to each patient. (**f**) The expression levels of subtype markers within each patient, including ESR1 and PGR for ER+, ERBB2 for HER2+, and VIM for TNBC subtypes. (**g**) PCA analysis of expression profiles from all patients, colored by subtypes. (**h**) Percentage distribution of molecular subtypes (LumA, LumB, Her2, Basal, and Normal) assigned to each patient. (**i**) Spearman correlation coefficient indicating the relationship between CCSs and each molecular subtype.

For Bhupinder_2021^39^ cohort, based on the clustering result (Fig. 4a and Supplementary Fig. 8a), we retrieved 28,615 malignant cells from 13 ER+, 6 HER2+ and 6 TNBC patients using CopyKAT^40^. Integrated analysis further divided all malignant cells into 13 clusters (C0-C12, Fig. 4b,c), in which C4, C6 and C10 formed a separate group and correspond to hc3 (Supplementary Fig. 8b). C9 and C5 highly expressed gene signatures of hc7 and hc10, respectively. When cells were ordered by clinical subtype, we found that signature genes of hc0, hc1 and hc4 showed higher expression in ER+ patients, those of hc2 were enriched in HER2+ patients while those of hc3 and hc10 were expressed in a part of cells of each subtype (Fig. 4d and Supplementary Fig. 8c). To confirm the CCS distribution in each subtype, we assigned each cell a CCS based on expression score of corresponding signatures. This analysis suggested that ER+ patients enriched for hc0, hc1 and hc4 except for ER0167 and ER0319, while HER2+ /TNBC patients enriched for hc2, hc3 and hc7, except for HER20161 (Fig. 4e). Although patients ER0167 and ER0319 were labeled ER positive, their cellular state compositions resembled those of HER2+ /TNBC patients. This promoted us to explore the expression levels of markers for ER+ (ESR1, PGR), HER2+ (ERBB2) and TNBC (VIM) subtype within patients. Interestingly, both ER0167 and ER0319 expressed comparable expression level of VIM with TNBC patients (Fig. 4f), however, ER0319 showed few expression of ERBB2 and almost no expression of ESR1 and PGR, and ER0167 also expressed ESR1. These observations implied the tendency to HER2+ /TNBC subtypes of two patients. PCA analysis further demonstrated the similarity of ER0319 with TNBC patients and ER0167 with HER2+ patients (Fig. 4g). Moreover, HER2+ patient HER20161 showed ER-like state composition and expressed not only ERBB2 but also ESR1 and PGR, suggesting its transformation from ER+ subtype (Fig. 4e–g). PAM50 analysis supported the correspondence between CCS and molecular subtype (Fig. 4h,i). Notably, ER0319 enriched “Normal” and “Basal” cells, highly similar with TNBC patients (Fig. 4g).

For Liu_2022^41^ and Xu_2021^42^ cohorts, the same analysis as above revealed similar results (Supplementary Figs. 9–S12). Specially, although patient_3 in Xu_2021 cohort was labeled HER2+, her state composition resembled ER+ patients since most cells were assigned hc4 state (Supplementary Fig. 11d). Notably, ERBB2 was expressed at very low level but ESR1 and PGR were highly expressed (Supplementary Fig. 11f), implying the misclassification of this patient. Molecular subtype analysis also supported this finding (Supplementary Fig. 11h).

All these results suggested that CCSs were general across breast cancer patients and could help to make more accurate classification and further therapeutic strategy.

### Specific intercellular communication contribute to the consensus cancer cell states

**Fig. 5:**
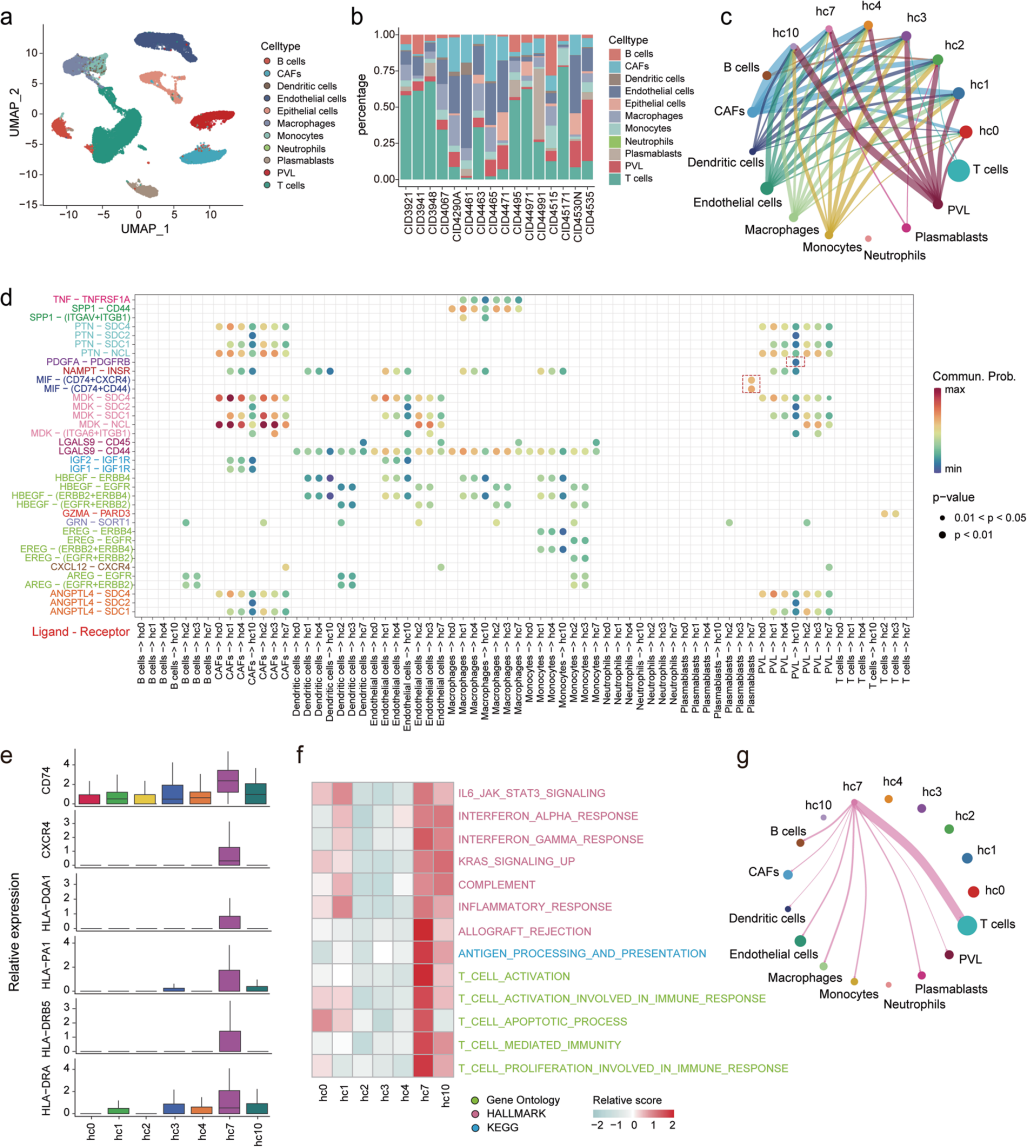
The cell-cell communication between CCSs and cell types in TME. (**a**) UMAP visualization of all non-malignant cells. (**b**) Percentage distribution of cell types across each patient. (**c**) Circular plots illustrating the inferred network connections among non-malignant cell types and CCSs. The edge width is proportional to the inferred communication amount. (**d**) Signal identification by comparative analysis of communication probabilities mediated by ligand-receptor pairs between non-malignant cell types and CCSs. (**e**) Expression of genes specific for hc7 that were involved in antigen presentation. (**f**) Heatmap showing the activities of immune-related pathways in each CCS. Pathway information sourced from Gene Ontology, MSigDB (hallmark), and KEGG. (**g**) Circular Plots displaying the strength of interactions from hc7 to all non-malignant cell types.

Breast cancer is characterized by distinct cellular microenvironments, where various non-tumorous cells exert significantly varied influences on tumor initiation and progression, therefore, we employed CellChat^43^ to explore the impact of the tumor microenvironment (TME) on CCSs. Among the 11 cell types in TME (Fig. 5a,b), cancer-related fibroblasts (CAFs), perivascular-like cells (PVLs) and endothelial cells exhibited substantial cellular communication with each CCS (Fig. 5c and Supplementary Fig. 13a). A majority of immune cells (dendritic cells, macrophages and monocytes) interacted with all CCSs via the LGALS9-CD44 ligand-receptor pair (Fig. 5d). Similarly, CAFs and PVL engaged with CCSs through ligand-receptor pairs involving PTN signaling (PTN-SDC4 and PTN-NCL), MDK signaling (MDK-SDC4) and ANGPTL signaling (ANGPTL4-SDC4) pathways. This suggested that SDC4 and NCL played a fundamental role in cancer cellular processes or were involved in regulatory mechanisms that were essential for various cell states. Evidently, unique signaling pathways underlined the influences of distinct microenvironmental cell types on tumor cells (Supplementary Figs. 13b, 14a). The VISFATIN and IGF signaling exclusively occurred in interactions between non-malignant cell types and hc1, hc4, and hc10. Similarly, the GRN signaling uniquely appeared in interactions with hc2. Moreover, the CXCR and MIF signaling pathways specifically manifested in interactions with hc7, and the PDGF signaling pathway exclusively emerged in interactions with hc10. Interestingly, in EGF signaling, hc1, hc4, and hc10 interacted with non-malignant cell types using ERBB4 and ERBB2+ ERBB4 complexes as receptors, while hc2 and hc3 communicated with the same cell types using EGFR and EGFR+ ERBB2 complexes as receptors. This observation might reflect the mechanism of the same cell types shaping the subtype-specific cancer cell states.

Apart from the common interactions, we also discerned a distinctive pattern where plasmablasts engaged exclusively with hc7 through ligand-receptor pairs MIF-(CD74 + CXCR4) and MIF- (CD74 + CD44) (Fig. 5d and Supplementary Fig. 14b). Macrophage migration inhibitory factor (MIF), a widely expressed cytokine, stimulates immune and epithelial cell proliferation, migration, and survival pathways through CD74^44^. CD74 is implicated in immune cell activation signaling, playing a pivotal role in histocompatibility complex II (MHCII)-restricted antigen presentation and anti-tumor immunity^45^. Consistently, MHCII-related genes were overexpressed in hc7 (Fig. 5e and Supplementary Fig. 14c) and known immunological pathways were enriched in hc7 (Fig. 5f), implying that plasmablasts potentially facilitated the presentation of exogenous antigens to T lymphocytes via interaction with the CD74 receptor on the surface of hc7 cells, thereby eliciting an immune-activated response. Accordingly, hc7 exhibited the most pronounced interaction signal strength with T cells (Fig. 5g and Supplementary Fig. 14d). Moreover, we observed distinct interactions between stromal cells (CAFs, endothelial cells, and PVL) and hc10 through the receptor SDC2 (Fig. 5d). The SDC2 downstream genes in the pathway of proteoglycans in cancer also showed relatively high expression levels in hc10 (Supplementary Fig. 14e). SDC2 has been demonstrated to contribute to cellular morphological changes, focal adhesions, and motility in breast cancer, ultimately inducing cell migration^46^. This alignment with the results of functional enrichment for hc10 supported the notion that hc10 might possess characteristics indicative of epithelial-mesenchymal transition (EMT), invasion and metastasis.

### Patients enriched for immune-related state (hc7) showed better outcome

**Fig. 6:**
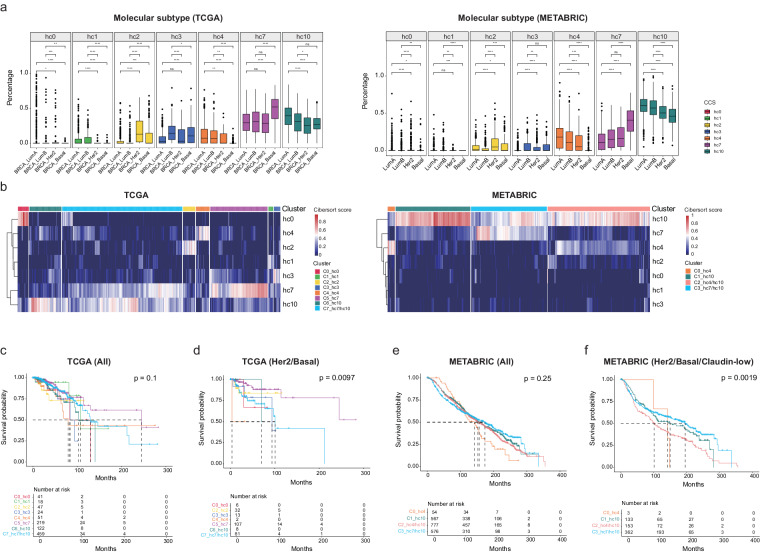
Deconvolution of breast cancer cohorts through CCS signatures unveils molecular subtype associations and patient survival. (**a**) Histograms depicting the distribution of each CCS within the four molecular subtypes (LumA, LumB, Her2, and Basal) across two breast cancer cohorts (TCGA and METABRIC). P values were calculated by a two-sided Wilcoxon rank-sum test (P-values denoted by asterisks: *p < 0.05, **p < 0.01, ***p < 0.001 and ****p < 0.0001.). (**b**) Clustering of all samples (columns) within the TCGA cohort (left) and the METABRIC cohort (right) showing 8 and 4 CCS-related clusters respectively. (**c**–**f**) Kaplan-Meier plots illustrating survival outcomes: overall TCGA cohort (**c**), TCGA subsets with Her2 or Basal subtypes (**d**), overall METABRIC cohort (**e**), and METABRIC subsets with Her2, Basal, or claudin-low subtypes (**f**). P-values were calculated using the log-rank test.

To investigate the association of consensus cancer cell states with the clinical metadata, we deconvoluted breast cancer profiles of the TCGA and METABRIC cohorts using CIBERSORT algorithm^47^ (see Methods), respectively. Molecular subtype results revealed that, in both cohorts, the percentages of hc0, hc1, hc4 and hc10 were higher in LumA and LumB subtypes while those of the other states were higher in Her2 subtype (hc2) or Basal subtype (hc3 and hc7, Fig. 6a). Consistent results were observed according to clinical subtype in METABRIC cohort (Supplementary Fig. 15a). Older women (aged more than 50 years) were correlated with significant increases in hc0 and hc4 and decreases in hc7 (Supplementary Fig. 15a).

Based on the CCS frequencies, all of cases were divided into clusters corresponding to each state or mixed state (Fig. 6b). Then we performed survival analysis of these tumor clusters either globally or in a subtype-specific manner. In the global analysis (using all patients with all molecular subtypes) of TCGA cohort, C5 (designated hc7) showed better outcome, followed by C7 with mixed states of hc7 and hc10 (Fig. 6c). In subtype-wise comparisons (using patients with Her2/Basal/Claudin-low subtypes), patients with high percentage of hc7 state displayed significantly longer survival period than others (Fig. 6d). In METABRIC cohort, although no patients with hc7 as the only predominant state, C3 with mixed states of hc7 and hc10 showed better outcome than the others, in both global and subtype-wise comparisons (Fig. 6e,f). These observations seem to imply the association of hc7 with good survival. To confirm this, patients were divided into two groups based on the cell percentage of hc7 state. In Her2/Basal subtypes, patients enriched for cells with hc7 state had a significantly longer survival time, in both TCGA and METABRIC cohorts (Supplementary Fig. 15b,c). Similar results were observed in a global analysis of TCGA cohort but not METABRIC cohort.

## Discussion

In this study, we revealed seven recurring breast cancer cell states with distinct functions by integrated analysis. These cancer cell states showed different cells of origin and close relation with breast cancer subtypes, including clinical and molecular subtypes, which may permit better classification of breast cancer patients.

Unlike previous studies that define cell states by identifying gene modules across cancer types^48,49^ or dissect the heterogeneity of cancer cells using few samples with small number of cells^50^, we focused on the breast cancer cells and took advantage of the batch correction method to remove batch effect from individual variation for data integration. This permitted us to handle large datasets and directly determine consensus cancer cell states of breast cancer, effectively eliminating the influence of intertumoral heterogeneity. The results revealed cancer cell states including hormone response (hc0), protein folding (hc1), lipid metabolism (hc2), proliferation (hc3), immune (hc5-hc7), hypoxia (hc8) and EMT (hc10). Although functional annotations of hc4 and hc9 were failed, the hallmark analysis suggested that hc9 was similar with hc2 and hc8 and may be involved in metabolism progresses, such as bile acid and cholesterol metabolism, while hc4 resembled hc0 and hc1 that may participate in estrogen and androgen response (Fig. 2f). Notably, hypoxia has been reported to be commonly associated with cancer, including shaping tumor microenvironment, promoting tumor progression and conferring resistance to conventional therapies^51,52^. However, hc8, the state associated with hypoxia and energy metabolism, showed an individual-specific pattern that it only enriched in one TNBC patient. Although the dataset we used contained abundant cells, more samples and cells may be required to perform deeper exploration of the universality or individuality of hypoxia state in breast cancer. Moreover, we also observed that hc5 and hc6, showing higher antigen processing and presentation pathway activities, only enriched in one TNBC patient (CID4515), suggesting strong crosstalk between tumor cells and microenvironment.

Among the seven consensus cancer cell states, although hc0, hc1, hc4 and hc10 showed close correlation, lineage analysis revealed their different cell of origin, as hc0, hc1 and hc4 may originate from mature luminal cells while hc10 may originate from basal/stem cells. Hc0, hc1 and hc4 were enriched in ER+ patients which are predominately composed of LumA and LumB subtypes, consistent with previous study that luminal-A/B breast cancers likely originate from mature luminal cells^29^. Given that the number of cells with hc10 state were relatively small but they existed in patients without subtype-tendency, we deduced that hc10 represents transient state between luminal and basal phenotypes. This was supported by the observations that although hc10 showed the expression similarity with luminal cells, the higher expression of mesenchymal and basal markers like VIM, COL1A2, ACTA2 and FN1 also indicated another identity of hc10. Consistently, the functional enrichment and hallmark analyses of hc10 suggested its relation with EMT, angiogenesis, extracellular matrix and collagen metabolism, which are frequently reported to be mediated by mesenchymal cells^53^ and commonly associated with caner progression^54^. However, hc2, hc3 and hc7 were enriched in HER2+ /TNBC patients, which was coincident with their cells of origin and previous reports that luminal progenitors are the source of basal-like breast cancers^23,55^ and HER2+ those may originate from luminal progenitors^29^. Cells with these three states also displayed higher differentiation potential than the others. Notably, the association between CCSs and subtypes can help to more accurately classify patients. For example, one patient (ER0319) in Bhupinder_2021 dataset was considered as ER+ subtype, however, we speculated that this patient should be TNBC subtype based on the CCS composition and low expression of luminal markers as well as high expression basal markers. All above results were validated in another three independent datasets, suggesting the reproducibility and clinical value of identified CCSs.

Since cancers greatly depend on the tumor microenvironment, which plays critical roles in each stage of cancer progression, from occurrence, progression, invasion and metastatic dissemination^56,57^, we explored the external factors shaping the consensus cancer cell states in TME, apart from the identified TFs. Although we observed close and similar interactions between diverse cell types in TME and tumor cells, distinct receptor-ligand complexes were involved for cells with different states. For example, HBEGF-ERBB4 were one of the interaction complexes responsible for the association of dendritic cells, endothelial cells, macrophages and monocytes with hc1, hc4 and hc10, while HBEGF-EGFR were employed in the interaction with hc2 and hc3 (Fig. 5d). To some extent, this may explain why hc1, hc4 and hc10 were enriched in ER+ patients while hc2 and hc3 tended to be predominant in HER2+ /TNBC patients. Besides, we also found unique receptor-ligand complexes participating in the cell-cell communications of specific cell type and CCS. Hc7 is an immune-associated state and the interactions with plasmablasts were mediated by the specific complexes MIF-(CD74 + CXCR4) and MIF-(CD74 + CD44), suggesting the particular role of plasmablasts in the formation of hc7 state. Similar phenomenon was observed in the interaction between PVL and cells with hc10 state by PDGFA-PDGFRB complexes (Supplementary Fig. 14f), implying the importance of PVL in shaping this CCS with cancer progression-associated biological functions, such as angiogenesis, EMT and extracellular matrix organization.

In summary, we present evidence of seven consensus cancer cell states that play diverse roles in cancer initiation and progression, and clarified the internal and external mechanisms of state shaping. Further investigations using larger size of multi-omics datasets to determine whether more elaborate cancer cell states exist, how these states distribute spatially in a tumor and what is the dynamics of the states along cancer progression, are warranted. Our study revealed the relation of the consensus cancer cell states with breast cancer subtypes and clinical outcome, which can aid in deepening our understanding of breast cancer cell heterogeneity, precisely determining the subtype identities of patients and developing individualized therapeutic strategy based on the state composition.

## Methods

### Data acquisition

The publicly available datasets of human breast cancer were downloaded, including four scRNA-seq cohorts and two bulk cohorts. The scRNA-seq datasets contained Wu_2021^17^, Liu_2022^41^, Xu_2021^42^ and Bhupinder_2021^39^. The bulk datasets contained TCGA^22^ and METABRIC^58,59^ cohorts.

Four scRNA-seq datasets were obtained and preprocessed as follows. The Wu_2021 was downloaded from the Gene Expression Omnibus (GEO) database (GSE176078), containing 26 primary tumors with three major clinical subtypes of breast cancer, including 11 ER+, 5 HER2+ and 10 TNBC patients. Treated patients may undergo alterations in the biological features of tumor cells, potentially influencing the elucidation and validation of the tumor state. Therefore, we removed 5 treated patients (CID3963, CID4066, CID4398, CID4513, and CID4523) from our analysis for a clearer and more accurate assessment of the intrinsic tumor characteristics. The Liu_2022 was downloaded from the GEO database (GSE167036), which contains 8 treatment-naïve breast cancer patients with lymph node metastases. Among the patients, 5 cases were luminal subtypes and 3 cases were the HER2+ subtype. The Xu_2021 was downloaded from the GEO database (GSE180286), comprising 1 luminal patient, 2 HER2-enriched patients and 2 TNBC patients. The Bhupinder_2021 was downloaded from the GEO database (GSE161529), containing 421,761 cells from 52 patients. We retrieved expression profiles of 26 tumors with ER+, HER2+ or TNBC subtypes. For Liu_2022 and Xu_2021, those from primary tumor samples were used for the downstream analysis.

The Wu_2021, Liu_2022, and Bhupinder_2021 were generated using 10x Genomics technologies. Cell Ranger pipeline was employed to process raw sequencing data, map to the reference genome GRCh38 and generate feature-barcode matrices. Another dataset (Xu_2021) was prepared using the Singleron Biotechnologies library preparation approach. Raw sequencing data aligned to the human genome reference sequence (GRCH38) with STAR. The CeleScope pipeline was used to sample demultiplexing, barcode processing, and single-cell gene counting to generate a digital gene-cell matrix from this data (details see Supplementary Table 4).

The Wu_2021 dataset generated a total of 496.7 million reads with averaged depths of 7,590 reads per cell. Approximately 290, 918.3 and 121.2 million reads were respectively generated in Liu_2022, Bhupinder_2021 and Xu_2021, resulting in averaged depths of 5901, 4960 and 3975 reads per cell, respectively. The averaged coverages of 1891, 1613, 1335 and 1416 genes were detected per cell in Wu_2021, Liu_2022, Bhupinder_2021 and Xu_2021, respectively (more details see Supplementary Table 4). Given the higher unique molecular identifiers (UMIs) counts and number of detected genes, we used the Wu_2021 dataset for the main analysis and the others were utilized as validation datasets.

Two bulk datasets were both acquired from cBioPortal (https://www.cbioportal.org). The TCGA cohort contains 1082 tumor samples with survival status and follow-up information, among which 499 samples were LumA, 197 samples were LumB, 171 samples were Basal, 78 samples were Her2, 36 samples were Normal and 101 samples were unknown. We used only four subtypes for subsequent analyses, including LumA, LumB, Basal and Her2. The METABRIC cohort contains 1974 samples with clinical subtype information, including 700 LumA, 475 LumB, 209 Basal, 218 claudin-low, 224 Her2 and 148 Normal. The subsequent survival analysis was conducted on a total of 1826 samples, comprising five subtypes: LumA, LumB, Basal, Her2, and claudin-low.

### scRNA-seq data processing

For individual samples, the *EmptyDrops* method from the DropletUtils^60^ package (version 1.22.0) was applied to filter the raw UMIs count matrix for real barcodes from ambient background RNA cells, which indicated the absence of empty drops. The gene expression matrix was processed and analyzed by Seurat^61^ (version 4.3.0.1) package. For each dataset, we first excluded cells containing fewer than 200 genes or 500 UMIs. This step aimed to further remove cell debris, empty drops, and low-quality cells. Likely dying or apoptotic cells where more than 20% of transcripts derived from the mitochondria were also excluded. Following the initial clustering, we removed likely cell doublets and multiplets (see Doublet detection and removal). As described by previous research^62–64^, we excluded genes detected in fewer than five cells. All expression data were normalized with log transformation.

### Batch effect correction

For each dataset, we ran Harmony^21^ (version 0.1.1), the top batch mixing method with good performance^65^, to eliminate batch effects in the PCA space when clustering of major cell lineages before any clustering analysis or cell type annotation was performed. We meticulously assessed Harmony’s performance in terms of its capability to integrate data from multiple patients while maintaining cell type separation. Similarly, the expression profiles of all malignant cells were integrated using Harmony, which can eliminate batch effects from the heterogeneity between cancer cells of individual patient in each dataset.

### Cell clustering and cell type annotation

Seurat was applied to first normalize gene expression count data by the *NormalizeData* and *ScaleData* functions. Then *FindVariableFeatures* function was applied to select the top 2000 variable genes and principal component analysis (PCA) was performed. Cell clusters were identified using the *FindNeighbors* and *FindClusters* functions. To annotate cell clusters, the following procedure was used: (1) In the Wu_2021 dataset, we leveraged the available annotations and utilized the SingleR^18^ package (version 2.0.0) to assign cell types to each cell, including epithelial cells, B cells, plasmablasts, T cells, cancer-related fibroblasts (CAF), perivascular-like cells (PVL), endothelial cells, dendritic cells, macrophages, monocytes and neutrophils; (2) For three additional validation datasets, we scored each cluster by the normalized expressions of the following canonical markers: epithelial cells (EPCAM, KRT18, KRT7, KRT8), T cells (CD3D, CD3E, CD3G), B cells (CD79A, CD79B, CD19, MS4A1), plasmablasts (JCHAIN, MZB1), myeloid (LYZ, CD68, FCGR3A, MARCO), CAFs (COL1A1, DCN, COL1A2, C1R), smooth muscle cells (ACTA2) and endothelial cells (PECAM1, VWF, CLDN5, CDH5, FLT1, RAMP2). The highest scored cell type was assigned to each cluster.

### Doublet detection and removal

Likely doublets were identified and meticulously removed using a multi-step approach. Specifically, the following methods were employed to identify doublets: (1) Doublet detection algorithms: we applied the *findDoubletClusters* function supported in the scDblFinder^66^ (version 1.16.0) package to predict potential doublet clusters. (2) Cluster marker gene expression: cells of a cluster co-expressing discrepant lineage markers were identified and removed. We carefully checked the expression levels and proportions of canonical lineage-related marker genes in each scDblFinder identified doublet clusters to ensure the exclusion of most barcodes associated with cell doublets.

### Identifying malignant cells from breast epithelial cells

In the Wu_2021 dataset, we utilized InferCNV^19^ (version 1.14.2) to infer large-scale chromosomal copy number variations (CNVs) from the scRNA-seq data of epithelial cells. As controls for the CNV analysis, immune and stromal cells from the same dataset were selected. The degree of chromosomal copy number variation and the clustering distribution of cells allowed us to infer the malignancy of cancer cells.

In the validation datasets, since we aimed to confirm the widespread presence of CCSs as well as their characteristics in tumor cells without clone identification, we used the CopyKAT^40^ package (version 1.1.0) to distinguish tumor cells from normal epithelial cells as it did not require controls and showed a strong accuracy. By analyzing single-cell transcriptome data, CopyKAT infers cell ploidy, classifying cells as either diploid for normal cells or aneuploid for tumor cells. Based on the results from these two tools, we removed 5 patients without malignant cells (CID4040, CID3586, CID3838, CID33946 and CID44041) in Wu_2021 dataset, one patient (CA3) with only 2 malignant cells in Liu_2022 dataset and one patient (TN0106) with only 6 malignant cells in Bhupinder_2021 dataset for downstream analysis. The information of samples in this study was shown in Supplementary Table 1.

### Clone identification and association analysis of transcriptomic and genomic heterogeneity

In each patient, we performed cluster analysis using the inferCNV package with parameters HMM = T and hclust_method = ward. D2 to identify subclonal architecture. Consequently, cells in the same subclone exhibited identical copy number variations. Based on the transcriptomic cluster label and subclone label for each cell, we used the adjusted Rand index^20^ to compare the consistency between the two clustering results, thereby quantifying the correlation of heterogeneity at the transcriptomic and genomic levels. Subsequently, we defined subclone-specific CNV genes as those that exhibited unique CNV patterns or varying degrees of variation among subclones from inferCNV results. Moreover, we also employed the *FindAllMarkers* function to compute differential expression genes for each subclone. We measured the similarity between CNV genes and differential expression genes using the Jaccard similarity coefficient.

### Identifying consensus cell states (CCS) in breast cancer

Cell clusters were then identified using the *FindNeighbors* and *FindClusters* functions with a resolution set to 0.4. For visualization, clustering was performed by using the t-Distributed Stochastic Neighbor Embedding (t-SNE) or Uniform Manifold Approximation and Projection (UMAP) methods with Seurat functions *RunTSNE* and *RunUMAP*, respectively. We further identified differentially expressed genes (DEGs) as the signature for each cluster using the Wilcoxon test, which was implemented through the *FindAllMarkers* function. Genes were considered significant if they exhibited an average natural logarithm (fold change) of at least 1 and a Bonferroni-adjusted P-value less than 0.05. DEGs were further subjected to gene ontology (GO) enrichment analysis using the clusterProfiler^67^ (version 4.6.2 package with qvalueCutoff = 0.05. Fifty hallmark gene sets were downloaded from the Molecular Signature Database^68^ (MSigDB), and hallmark pathway scores were calculated for each cell using the *AddModuleScore* function.

### Evaluating cell stemness and differentiation potential

CytoTRACE^26^ (version 0.3.3) and SCENT^27^ (version 1.0.3) were applied to estimate the differentiation potential of each malignant cell based on their gene expression profiles. The CytoTRACE scores were ranked and scaled between 0 and 1, representing the predicted order of relative differentiation states of cells (0 for higher differentiation; 1 for lower differentiation). SCENT returned a vector where higher values indicated higher cellular differentiation potential, reflecting lower differentiation states and correspondingly higher cell stemness.

### Trajectory analysis

Using Monocle 3^28^ (version 1.3.4) with default parameters, we inferred the differentiation trajectory reflecting the cellular state transition of the malignant cells from all patients. Initially, Seurat object was directly imported using the *new_cell_data_set* function. The function *cluster_cells* and *plot_cells* were used for unsupervised clustering and visualization of CCSs. The function *learn_graph* was run with default parameters to construct the trajectory graph.

### Cell-cycle state assignment

The cell-cycle state of each cell was determined based on the expression profile of cell-cycle-related signature genes by the *CellCycleScoring* function that is implemented in Seurat package. Then, we categorized cells belonging to the G2M or S phase as cycling cells, while cells in the G1 phase were classified as non-cycling cells.

### PAM50 analysis

Genefu^30^ (version 2.32.0) was used to perform molecular subtyping of breast cancer cells based on known classification genes, including lumA, LumB, Her2, Basal, and Normal subtypes. Initially, cells without expression of all classification genes were labeled as “not.defined”. We then employed ggplot2^69^ (version 3.4.2) to visualize the distribution of molecular subtypes across the CCSs. To assess the association between CCSs and molecular subtypes, we calculated the spearman correlation coefficient between the proportions of each molecular subtype and each CCS across patients in a pairwise manner. The results were further visualized using the pheatmap (version 1.0.12) package.

### Identification of differentially activated TFs in each CCS

We conducted single-cell regulatory network inference and clustering (SCENIC) analysis on all cells of CCSs, using R package SCENIC^31^ (version 1.3.1). Transcription factor (TF) searching regions were limited to a 10k distance centered around the transcriptional start site (TSS) or 500 bp upstream of the TSSs. First, SCENIC utilized GENIE3 (version 1.20.0) (a random forest-based method) to infer co-expression modules between transcription factors and candidate target genes. Each module contained a transcription factor and its corresponding target genes. RcisTarget (version 1.18.2) was employed to analyze genes within each co-expression module, identifying enriched motifs. Each TF, along with its potential direct target genes, was referred to a regulon. Finally, AUCell (version 1.20.2) was used to assess the activity of each regulon in individual cell, generating a regulon activity matrix. By following these steps, we obtained the regulatory relationships between transcription factors and their target genes in a cellular context. ComplexHeatmap^70^ (version 2.14.0) was used to display regulon activity scores for each individual cell. The differentially activated TFs of each CCS were identified by the Wilcoxon rank sum test, and significantly upregulated TFs were identified with a logarithm fold change more than 1 and an FDR less than 0.05. The top 5 regulons specific for CCSs were shown in Supplementary Fig. 5d (only 3 regulons identified in hc4).

### ST data acquisition

Three spatial transcriptomics datasets were obtained from publicly available databases, including ST_Wu_2021^34^, ST_Angele_2023^35^, and ST_Amanda_2023^36^. The ST_Wu_2021 was downloaded from the Zenodo data repository, containing one ER+ samples (CID4535) and four TNBC samples (CID44971, 1142243 F, 1160920 F and CID4465). The ST_Angele_2023 was downloaded from the GEO database (GSE213688), containing 6 TNBC samples (M2, M5, M6, M11, M14 and M15). The ST_Amanda_2023 was downloaded from the GEO database (GSE243275), which contained one sample of breast cancer with HER2+ subtype. The pathology annotation for each spot was also downloaded. The spots of all samples from ST_Wu_2021 and ST_Angele_2023 were annotated by professional pathologists, while those of the sample from ST_Amanda_2023 were annotated by a supervised labeling method.

### ST data processing and identification of region-specific marker genes

For each spatial transcriptomics data, spots with less than 200 genes or mitochondrial transcripts greater than 20% were discarded, and genes expressed in less than 2 spots were removed. Count matrix was standardized with *SCTransform* function in Seurat package to account for variance in sequencing depth across data points and detect high-variance features. Based on the pathology annotation for tissue regions, we identified region-specific marker genes through *FindAllMarkers* function (logfc.threshold > 1 and p_val_adj < 0.05).

### Deconvolution analyses of spatial transcriptomics data

To investigate the spatial location of CCSs in breast cancer, we employed CARD^37^ (version 1.1) method to deconvolute the spatial transcriptomics data based on the single-cell data of CCSs. The CARD performed deconvolution through a non-negative factorization framework and output the estimated composition of CCSs across spatial locations with two inputs, which included the single-cell transcriptional profiles of all CCSs and the ST data with localization information. The *CARD_deconvolution* function with default parameters was utilized to calculate the proportion of CCSs at each spatial location.

### Multimodal intersection analysis (MIA)

We used MIA^38^, which performed hypergeometric cumulative distribution on the overlap between region-specific marker genes and the DEGs of CCSs, to further determine CCSs enrichment in tissue regions. All genes were used as the background to calculate the P-value.

### Cell-cell interaction using CellChat

In order to explore cell-cell interactions across different cell types including non-malignant cells and malignant cells with distinct CCS, we utilized CellChat^43^ (version 1.6.1) to identify significant ligand-receptor pairs with default parameters. The Seurat object containing both the count matrix and cell type labels for each individual cell was seamlessly integrated into the CellChat framework. Subsequently, we harnessed the power of the CellChatDB database to identify relevant ligand-receptor pairs. By projecting ligands and receptors onto a Protein-Protein Interaction (PPI) network, we calculated communication probabilities and inferred the intricate CellChat network. Furthermore, to ensure the accuracy of our findings, we filtered out the results of cell types with less than 10 cells.

### Principal components analysis

In the validation datasets, we computed the average expression of all cells for each sample, creating a pseudo-bulk dataset. Based on this data, PCA analysis was performed to explore the clustering patterns of different subtype samples. For visualization purposes and effective presentation of the results, ggplot2 was employed.

### Estimating the CCS composition in bulk samples and survival analysis

The CIBERSORT^47^ algorithm was strategically applied to deconvolute bulk expression data from TCGA and METABRIC cohorts and estimate relative CCS proportions within individual patient. Based on these inferred proportions, a comprehensive hierarchical clustering analysis was conducted to uncover inherent patterns and relationships among the samples. Furthermore, survival analysis was performed using the Survival package (version 3.4-0). Specifically, relevant clinical subtypes were selected to generate survival curves for patient overall survival (OS) and statistical testing. When focusing on one CCS, patient groups were divided based on the ‘median’ criterion, separating patients into lower and higher groups. This analysis delved into the intricate interplay between inferred CCSs proportions and sample survival outcomes, yielding insights into potential correlations between state composition and survival trends.

### Statistical analyses

In addition to the bioinformatics approaches described above for scRNA-seq data analysis, all other fundamental statistical analyses were performed in the R statistical environment. The assessment of differences in continuous variables, such as gene expression, regulon activities, and gene set scoring, between two groups (defined by a categorical independent variable, such as ER+ versus HER2+ subtype), was carried out using the Wilcoxon rank sum test. To control for multiple hypothesis testing, we applied the Bonferroni correction method to correct P values and the false discovery rates (FDR q-values) were calculated. Results were considered statistically significant at P-value or FDR q-value < 0.05.

### Supplementary information

Supplementary table 1

Supplementary table 2

Supplementary table 3

Supplementary table 4

Supplementary Information

## Data Availability

All the scRNA-seq data, spatial transcriptomics data and bulk RNA-seq data were downloaded from previous publications or public domains. The four scRNA-seq datasets were obtained from Gene Expression Omnibus (GEO, https://www.ncbi.nlm.nih.gov/geo) at GSE176078^17^, GSE167036^41^, GSE180286^42^, and GSE161529^39^. And two spatial transcriptomics datasets (ST_Angele_2023 and ST_Amanda_2023) were also obtained from GEO at GSE213688^35^ and GSE243275^36^. ST_Wu_2021 was acquired from Zenodo repository (10.5281/zenodo.4739739)^34^. Two bulk datasets were both acquired from cBioPortal at https://www.cbioportal.org. The processed data and intermediate results in our manuscript were uploaded on Figshare repository at 10.6084/m9.figshare.25019903^71^.

## Code availability

All codes used for analysis can be found on GitHub at https://github.com/pangbo2019/BRCA_CCS.

## References

[1] Sung H (2021). Global Cancer Statistics 2020: Globocan Estimates of Incidence and Mortality Worldwide for 36 Cancers in 185 Countries. CA Cancer J Clin..

[2] Nolan E, Lindeman GJ, Visvader JE (2023). Deciphering Breast Cancer: From Biology to the Clinic. Cell..

[3] Marusyk A, Janiszewska M, Polyak K (2020). Intratumor Heterogeneity: The Rosetta Stone of Therapy Resistance. Cancer Cell..

[4] Shaffer SM (2020). Memory Sequencing Reveals Heritable Single-Cell Gene Expression Programs Associated with Distinct Cellular Behaviors. Cell..

[5] Gambardella G (2022). A Single-Cell Analysis of Breast Cancer Cell Lines to Study Tumour Heterogeneity and Drug Response. Nat Commun..

[6] Wagner J (2019). A Single-Cell Atlas of the Tumor and Immune Ecosystem of Human Breast Cancer. Cell..

[7] Wu SZ (2021). A Single-Cell and Spatially Resolved Atlas of Human Breast Cancers. Nat Genet..

[8] Cheang MC (2015). Defining Breast Cancer Intrinsic Subtypes by Quantitative Receptor Expression. Oncologist..

[9] Perou CM (2000). Molecular Portraits of Human Breast Tumours. Nature..

[10] Sorlie T (2001). Gene Expression Patterns of Breast Carcinomas Distinguish Tumor Subclasses with Clinical Implications. Proc Natl Acad Sci USA.

[11] Nguyen QH (2018). Profiling Human Breast Epithelial Cells Using Single Cell Rna Sequencing Identifies Cell Diversity. Nat Commun..

[12] Pal B (2021). A Single-Cell Rna Expression Atlas of Normal, Preneoplastic and Tumorigenic States in the Human Breast. EMBO J..

[13] Bhat-Nakshatri P (2021). A Single-Cell Atlas of the Healthy Breast Tissues Reveals Clinically Relevant Clusters of Breast Epithelial Cells. Cell Rep Med..

[14] Kumar T (2023). A Spatially Resolved Single-Cell Genomic Atlas of the Adult Human Breast. Nature..

[15] Liu SQ (2022). Single-Cell and Spatially Resolved Analysis Uncovers Cell Heterogeneity of Breast Cancer. J Hematol Oncol..

[16] Hu L (2021). Single-Cell Rna Sequencing Reveals the Cellular Origin and Evolution of Breast Cancer in Brca1 Mutation Carriers. Cancer Res..

[17] Wu SZ (2021). GEO..

[18] Aran D (2019). Reference-Based Analysis of Lung Single-Cell Sequencing Reveals a Transitional Profibrotic Macrophage. Nat Immunol..

[19] Tirosh I (2016). Dissecting the Multicellular Ecosystem of Metastatic Melanoma by Single-Cell Rna-Seq. Science..

[20] Rand WM (1971). Objective Criteria for the Evaluation of Clustering Methods. Journal of the American Statistical Association..

[21] Korsunsky I (2019). Fast, Sensitive and Accurate Integration of Single-Cell Data with Harmony. Nat Methods..

[22] Hoadley KA (2018). Cell-of-Origin Patterns Dominate the Molecular Classification of 10,000 Tumors from 33 Types of Cancer. Cell..

[23] Lim E (2009). Aberrant Luminal Progenitors as the Candidate Target Population for Basal Tumor Development in Brca1 Mutation Carriers. Nat Med..

[24] Visvader JE, Stingl J (2014). Mammary Stem Cells and the Differentiation Hierarchy: Current Status and Perspectives. Genes Dev..

[25] Wang D (2015). Identification of Multipotent Mammary Stem Cells by Protein C Receptor Expression. Nature..

[26] Gulati GS (2020). Single-Cell Transcriptional Diversity Is a Hallmark of Developmental Potential. Science..

[27] Teschendorff AE, Enver T (2017). Single-Cell Entropy for Accurate Estimation of Differentiation Potency from a Cell’s Transcriptome. Nat Commun..

[28] Cao JY (2019). The Single-Cell Transcriptional Landscape of Mammalian Organogenesis. Nature..

[29] Prat A, Perou CM (2009). Mammary Development Meets Cancer Genomics. Nat Med..

[30] Gendoo DM (2016). Genefu: An R/Bioconductor Package for Computation of Gene Expression-Based Signatures in Breast Cancer. Bioinformatics..

[31] Aibar S (2017). Scenic: Single-Cell Regulatory Network Inference and Clustering. Nat Methods..

[32] Bommi-Reddy, A. *et al*. Efficacy of a Novel Ep300/Cbp Histone Acetyltransferase Inhibitor in Hormone Responsive Breast Cancer. *Cancer Research*. **79** (2019).

[33] Nicole Tsang YH (2013). Prolyl Isomerase Pin1 Downregulates Tumor Suppressor Runx3 in Breast Cancer. Oncogene..

[34] Wu SZ (2021). Zenodo..

[35] Coutant A (2023). GEO..

[36] Janesick A (2023). GEO.

[37] Ma Y, Zhou X (2022). Spatially Informed Cell-Type Deconvolution for Spatial Transcriptomics. Nature Biotechnology..

[38] Moncada R (2020). Integrating Microarray-Based Spatial Transcriptomics and Single-Cell Rna-Seq Reveals Tissue Architecture in Pancreatic Ductal Adenocarcinomas (Vol 38, Pg 333, 2020). Nature Biotechnology..

[39] Pal B (2021). GEO..

[40] Gao R (2021). Delineating Copy Number and Clonal Substructure in Human Tumors from Single-Cell Transcriptomes. Nat Biotechnol..

[41] Liu T, Liu C, Zhang J, Wei X, Zhang H (2022). GEO..

[42] Xu K (2021). GEO..

[43] Jin S (2021). Inference and Analysis of Cell-Cell Communication Using Cellchat. Nat Commun..

[44] Leng L (2003). Mif Signal Transduction Initiated by Binding to Cd74. J Exp Med..

[45] Schroder B (2016). The Multifaceted Roles of the Invariant Chain Cd74–More Than Just a Chaperone. Biochim Biophys Acta..

[46] Lim HC, Couchman JR (2014). Syndecan-2 Regulation of Morphology in Breast Carcinoma Cells Is Dependent on Rhogtpases. Biochim Biophys Acta..

[47] Newman AM (2015). Robust Enumeration of Cell Subsets from Tissue Expression Profiles. Nat Methods..

[48] Kinker GS (2020). Pan-Cancer Single-Cell Rna-Seq Identifies Recurring Programs of Cellular Heterogeneity. Nat Genet..

[49] Barkley D (2022). Cancer Cell States Recur across Tumor Types and Form Specific Interactions with the Tumor Microenvironment. Nat Genet..

[50] Chung W (2017). Single-Cell Rna-Seq Enables Comprehensive Tumour and Immune Cell Profiling in Primary Breast Cancer. Nat Commun..

[51] Riera-Domingo C (2020). Immunity, Hypoxia, and Metabolism-the Menage a Trois of Cancer: Implications for Immunotherapy. Physiol Rev..

[52] Jing X (2019). Role of Hypoxia in Cancer Therapy by Regulating the Tumor Microenvironment. Mol Cancer..

[53] Koliaraki V, Pallangyo CK, Greten FR, Kollias G (2017). Mesenchymal Cells in Colon Cancer. Gastroenterology..

[54] Pastushenko I, Blanpain C (2019). Emt Transition States During Tumor Progression and Metastasis. Trends Cell Biol..

[55] Proia TA (2011). Genetic Predisposition Directs Breast Cancer Phenotype by Dictating Progenitor Cell Fate. Cell Stem Cell..

[56] Wu F (2021). Signaling Pathways in Cancer-Associated Fibroblasts and Targeted Therapy for Cancer. Signal Transduct Target Ther..

[57] de Visser KE, Joyce JA (2023). The Evolving Tumor Microenvironment: From Cancer Initiation to Metastatic Outgrowth. Cancer Cell..

[58] Pereira B (2016). The Somatic Mutation Profiles of 2,433 Breast Cancers Refines Their Genomic and Transcriptomic Landscapes. Nat Commun..

[59] Curtis C (2012). The Genomic and Transcriptomic Architecture of 2,000 Breast Tumours Reveals Novel Subgroups. Nature..

[60] Lun, A. T. L. *et al*. Emptydrops: Distinguishing Cells from Empty Droplets in Droplet-Based Single-Cell Rna Sequencing Data. *Genome Biology*. **20** (2019). 10.1186/s13059-019-1662-y PMC6431044 30902100

[61] Hao Y (2021). Integrated Analysis of Multimodal Single-Cell Data. Cell..

[62] Ambrosi TH (2021). Aged Skeletal Stem Cells Generate an Inflammatory Degenerative Niche. Nature..

[63] Elmentaite R (2021). Cells of the Human Intestinal Tract Mapped across Space and Time. Nature..

[64] Gate D (2020). Clonally Expanded Cd8 T Cells Patrol the Cerebrospinal Fluid in Alzheimer’s Disease. Nature..

[65] Tran HTN (2020). A Benchmark of Batch-Effect Correction Methods for Single-Cell Rna Sequencing Data. Genome Biol..

[66] Germain, P.-L., Lun, A., Macnair, W., Robinson, M. D. Doublet Identification in Single-Cell Sequencing Data Using Scdblfinder. *F1000Research*. **10** (2021). 10.12688/f1000research.73600.1 PMC9204188 35814628

[67] Wu T (2021). Clusterprofiler 4.0: A Universal Enrichment Tool for Interpreting Omics Data. Innovation (Camb)..

[68] Liberzon A (2015). The Molecular Signatures Database (Msigdb) Hallmark Gene Set Collection. Cell Syst..

[69] Wickham, H., Wickham, H. Data Analysis: Springer; 2016.

[70] Gu Z, Eils R, Schlesner M (2016). Complex Heatmaps Reveal Patterns and Correlations in Multidimensional Genomic Data. Bioinformatics..

[71] Pang L (2024). figshare..

