# Supplementary material for: Single-cell integrative analysis reveals consensus cancer cell states and clinical relevance in breast cancer
Source: Sci Data. 2024 Mar 12;11:289. doi: 10.1038/s41597-024-03127-0 (PMC10933444; doi:10.1038/s41597-024-03127-0)
Supplement: Supplementary file 5 — Supplementary Information [file 41597_2024_3127_MOESM5_ESM.pdf]

Supplementary Information

Single-cell integrative analysis reveals consensus cancer cell states and clinical relevance in breast cancer

Lin Pang[#, 1, *], Fengyu Xiang[#, 1], Huan Yang[#, 1], Xinyue Shen[1], Ming Fang[1], Ran Li[1], Yongjin Long[1], Jiali Li[1], Yonghuan Yu[1], Bo Pang[1, *]

This supplementary information includes:

Supplementary Figure 1- Supplementary Figure 15 page 2-16

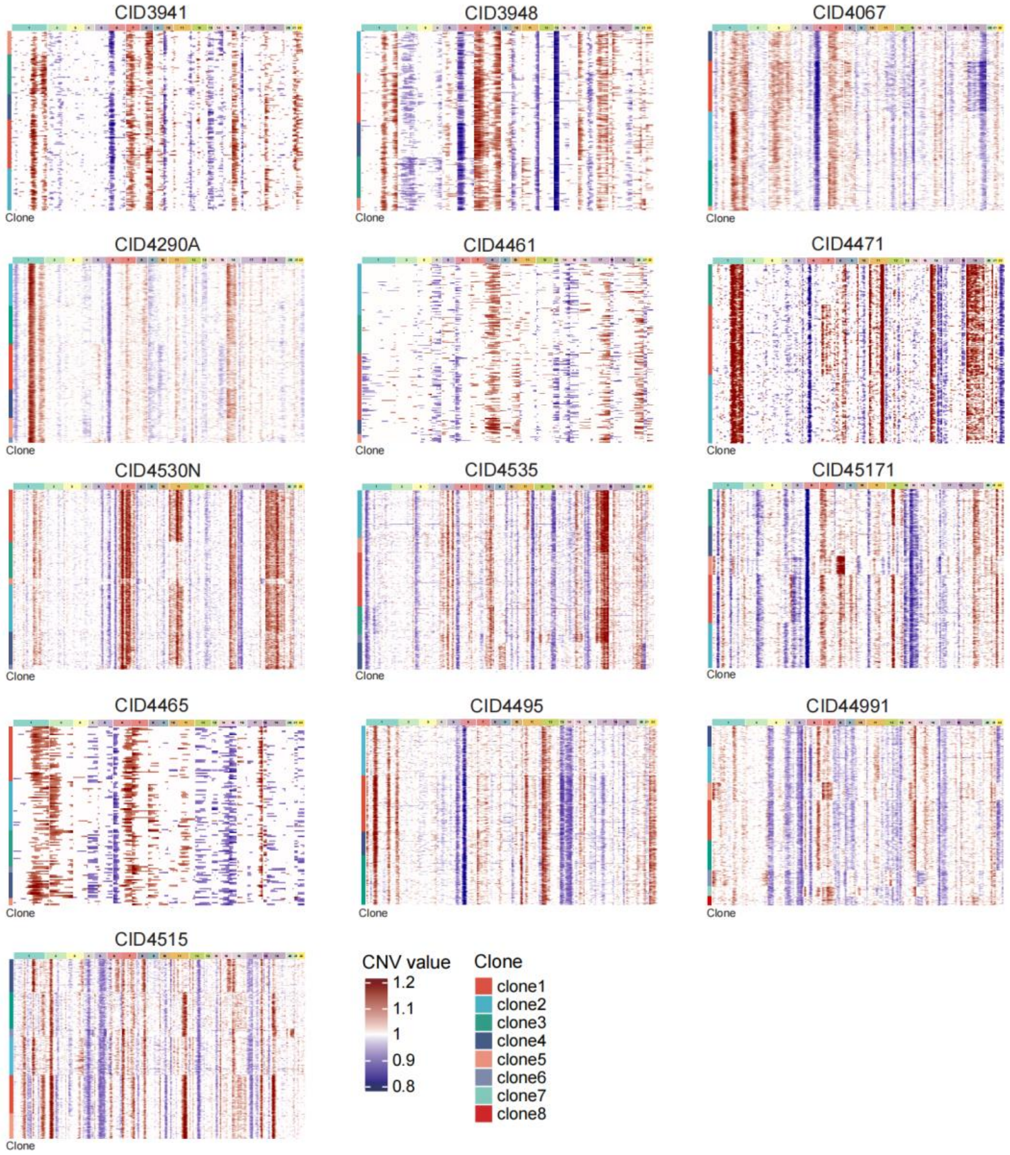

Supplementary Figure 1. The landscape of inferred large-scale CNVs for all of the tumor cells. InferCNV heatmaps display copy number scores for all malignant cells in 13 patients.

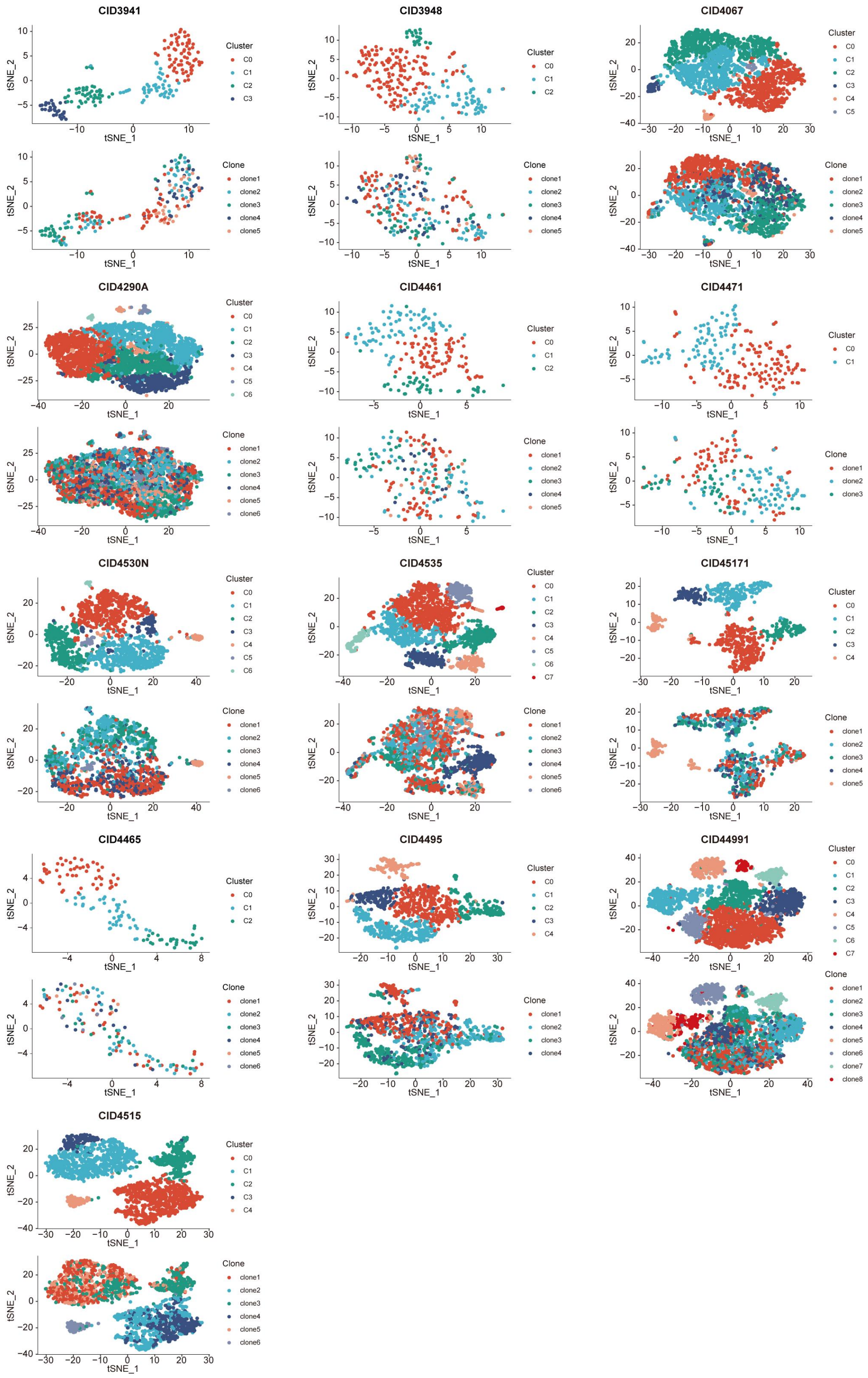

Supplementary Figure 2. Association map of transcriptomic and genomic heterogeneity of 13 patients. The upper scatter plot illustrates the transcriptome-based clustering of malignant cells. Cells were colored by clone label are visualized in the lower scatter plot.

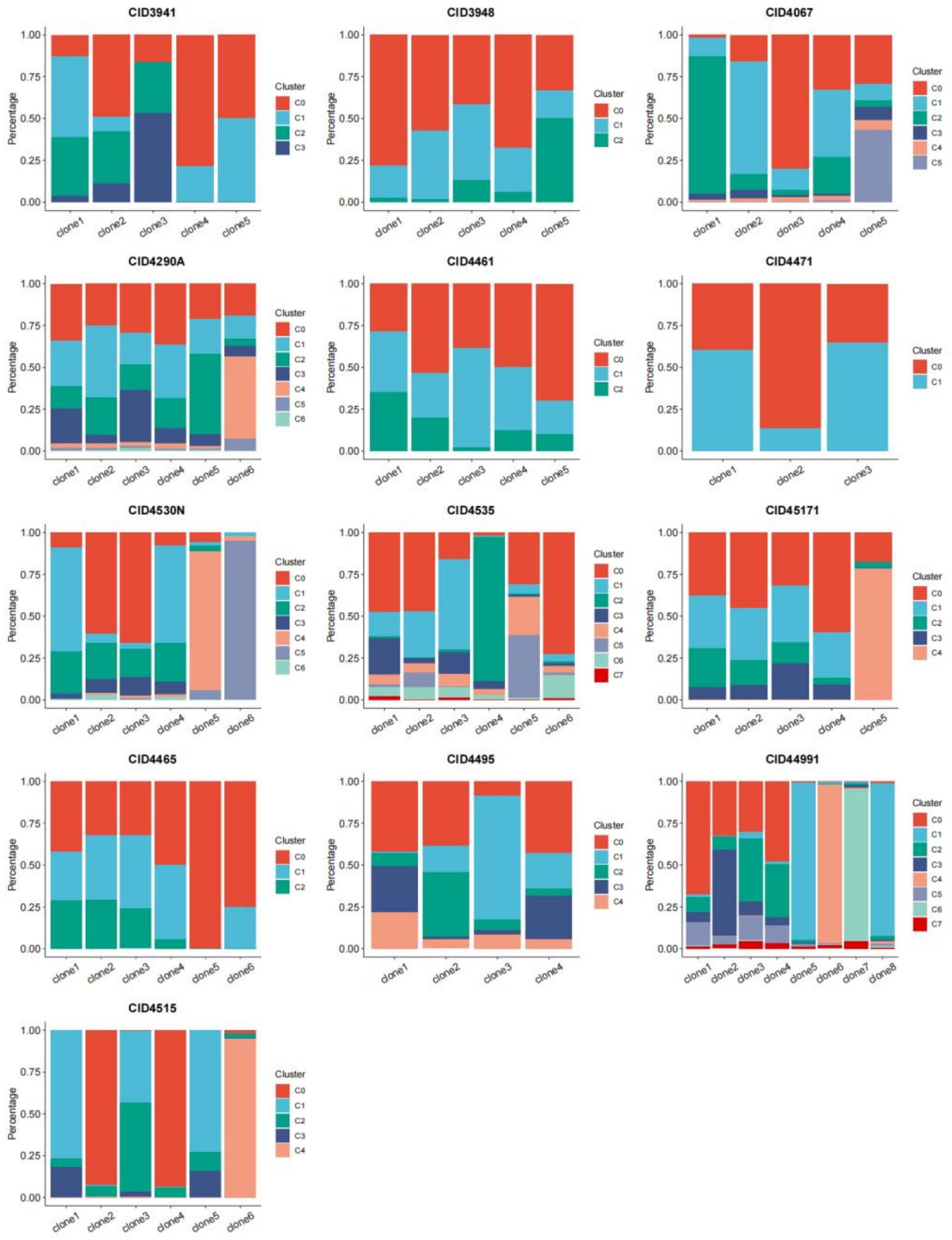

Supplementary Figure 3. Distribution of transcriptional clusters across subclones in 13 patients. The histograms showing the relative proportions of all clusters contained by each subclone for every patient.

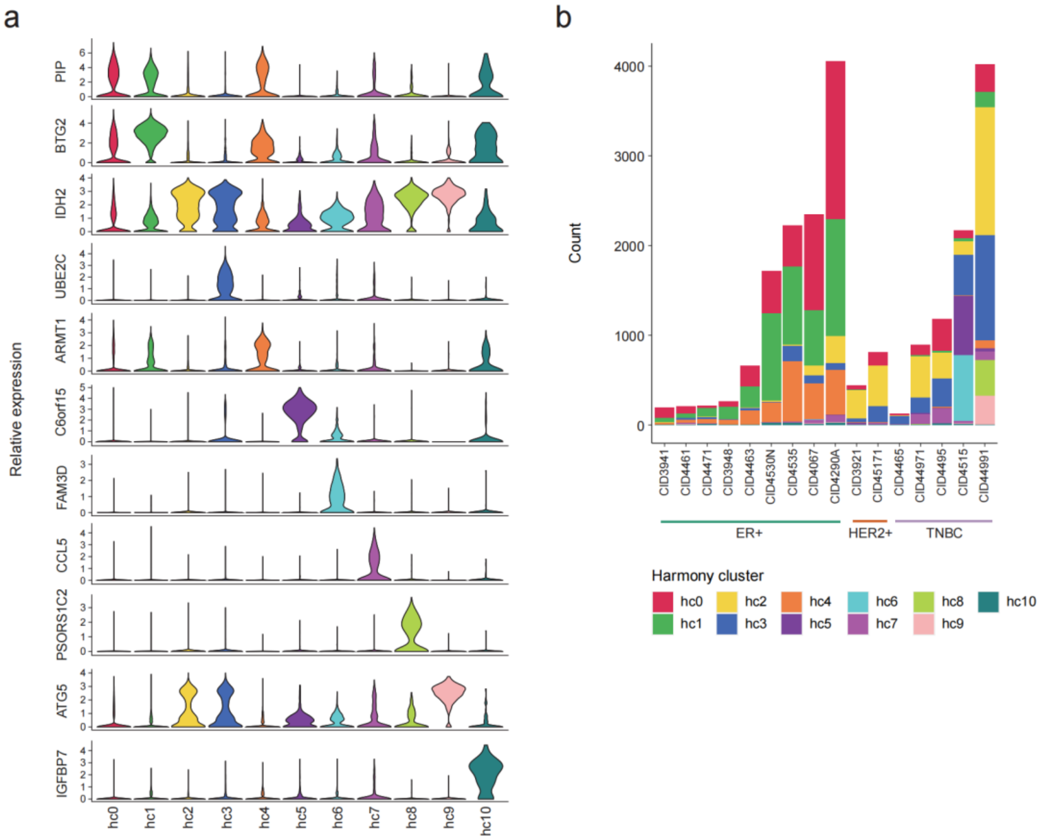

Supplementary Figure 4. Expression variations and distribution of harmony clusters. (a) Violin plot showing individual differentially expressed genes (adjusted p-value < 0.05 and log2 fold change > 1) for each harmony cluster. (b) Distribution of cell counts across 16 patients, colored by harmony clusters.

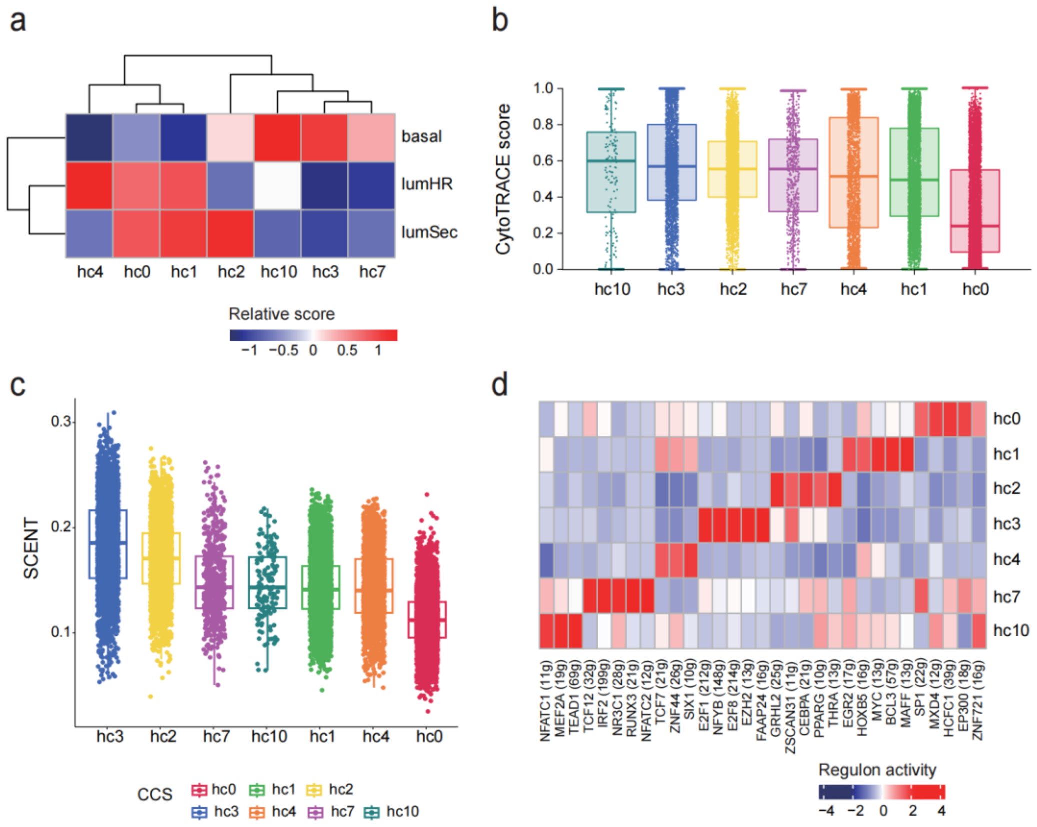

Supplementary Figure 5. Lineage origin, stemness, and unique regulons of CCSs. (a) Heatmap illustrating cellular lineage identity, encompassing basal, lumHR, and lumSec. (b-c) The differentiation potential of cells with CCSs evaluated by CytoTRACE (b) and SCENT (c) methods, respectively. (d) Heatmap highlighting the top 5 regulons that are specific to CCSs.

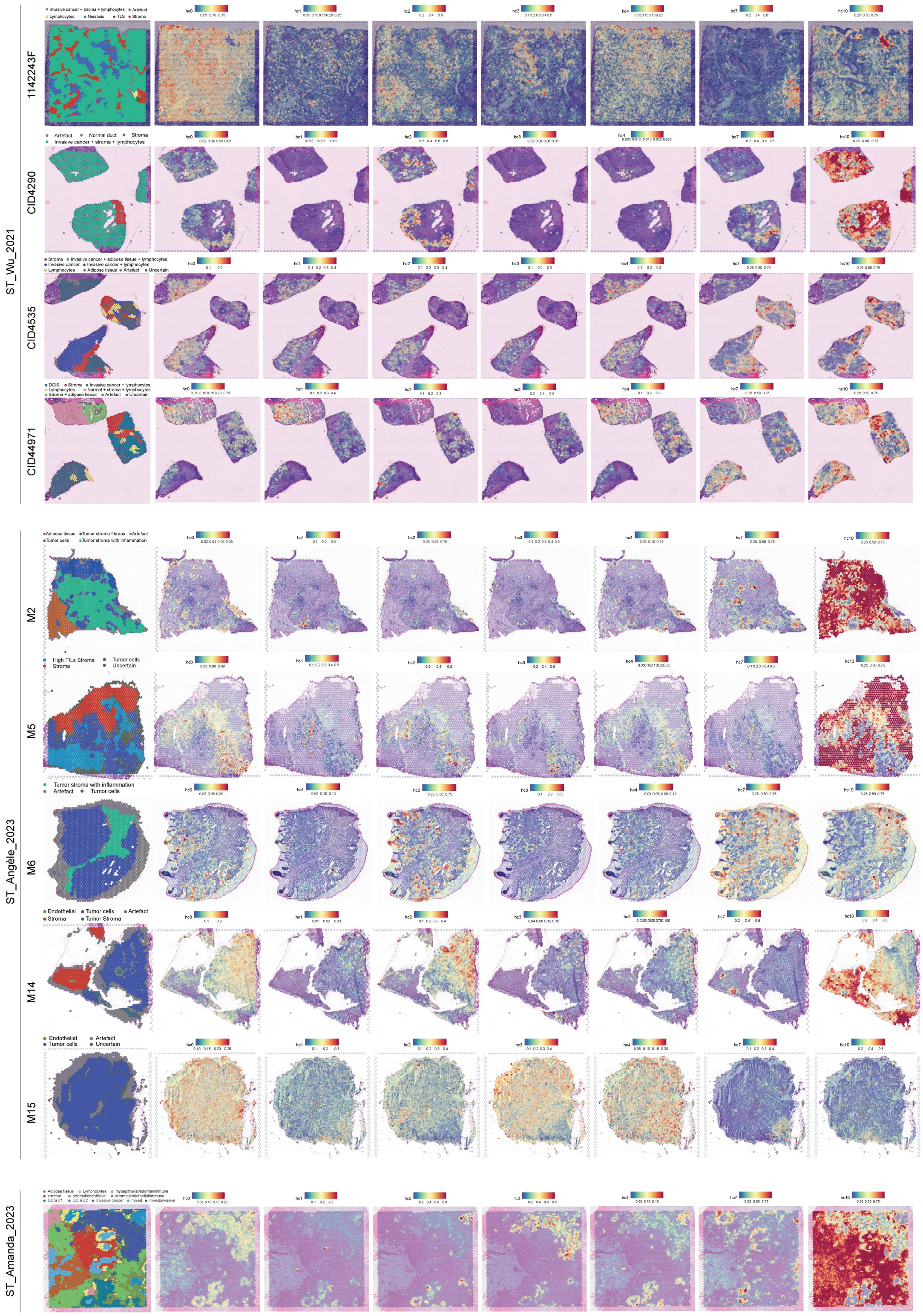

Supplementary Figure 6. Pathology annotations and Localization of the CCSs in ten patients from ST_Wu_2021, ST_Angele_2023, and ST_Amanda_2023 based on CARD. The proportion of CCSs for each spot was calculated using CARD. Red indicates a higher proportion, and blue indicates a lower proportion.

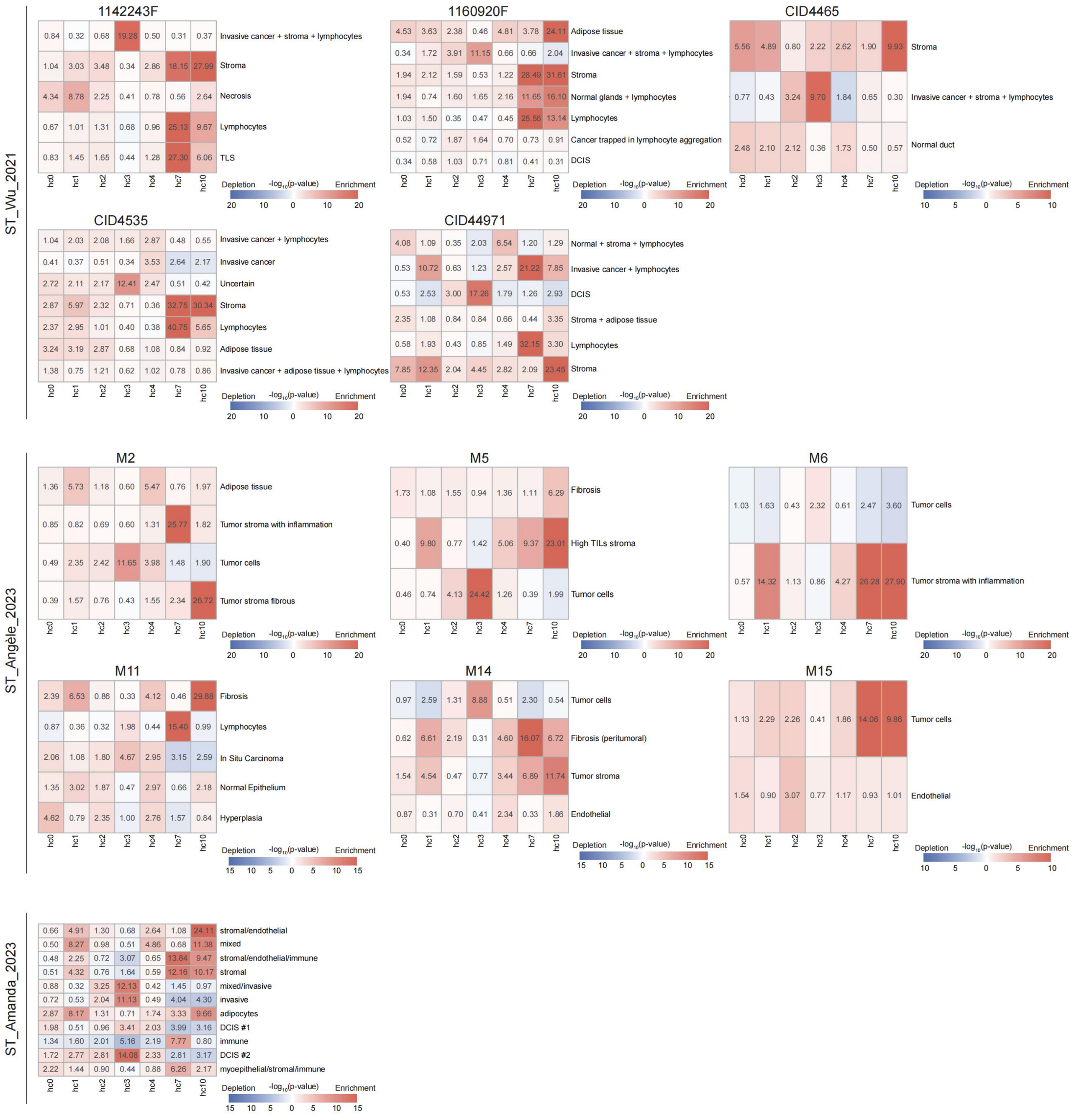

Supplementary Figure 7. MIA map of spatial transcriptomics regions and CCSs in 12 patients from ST_Wu_2021, ST_Angele_2023, and ST_Amanda_2023. Each element in the heatmap indicates the enrichment degree (-log10(p-value) of hypergeometric test) of CCSs in ST tissue regions, which are measured by testing on the overlap of their differential expression genes. Red indicates enrichment (significantly high overlap), and blue indicates depletion (significantly low overlap).

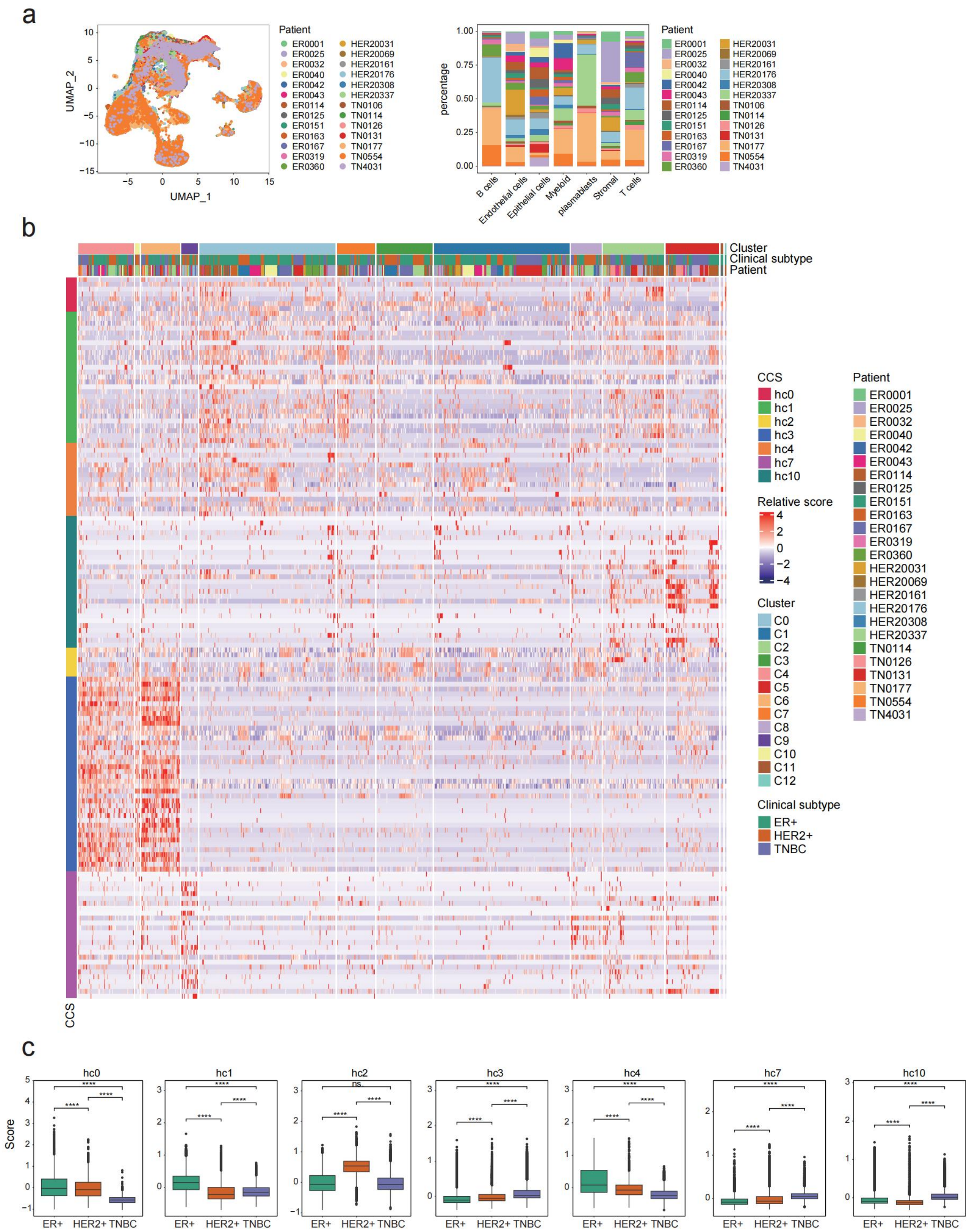

Supplementary Figure 8. Association of CCS with cell clusters and clinical subtypes in the Bhupinder_2021 cohort. (a) UMAP visualization of all cells (left) and distribution of cell counts across major cell types (right), colored by patients. (b) The expression levels of signature genes of CCSs within cancer cells of each cluster. (c) Box plots showing expression scores of signature genes of CCSs in the three clinical subtypes (ER+, HER2+, and TNBC). P values were determined through a two-sided Wilcoxon rank-sum test (*p < 0.05, **p < 0.01, ***p < 0.001, and ****p < 0.0001, denoted by asterisks).

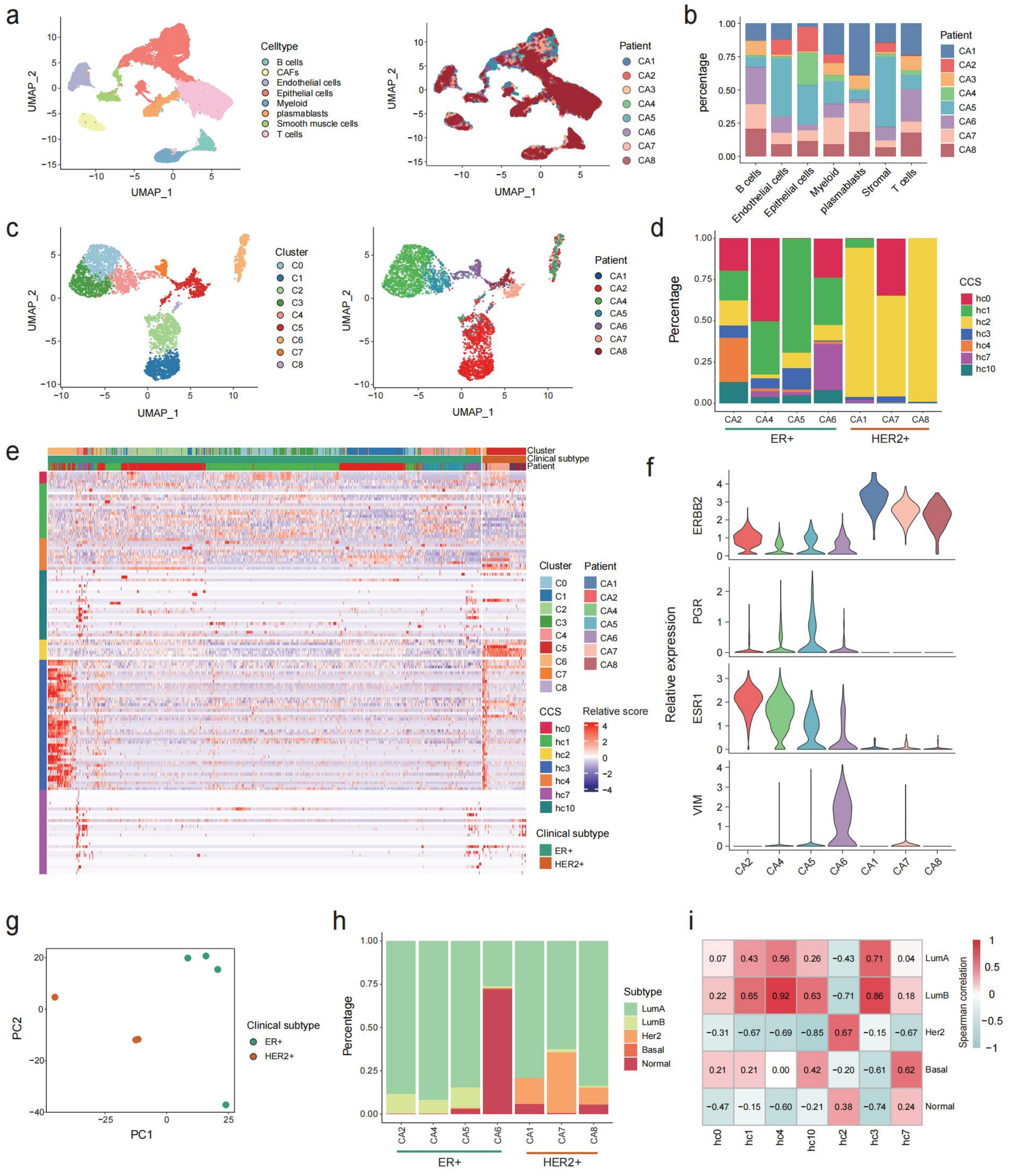

Supplementary Figure 9. The consistency verification of CCSs, clinical subtypes, and molecular subtypes in the Liu_2022 cohort. (a) UMAP visualization of all cells, colored by the major cell types annotated based on canonical markers (left), and patients (right). (b) The percentages of cells from all patients in each major cell type. (c) UMAP visualization of all malignant cells from patients with at least 10 malignant cells, colored by cluster (left) and patient (right). (d) Percentage distribution of CCSs assigned to each patient. (e) The expression levels of signature genes of CCSs within cancer cells of each clinical subtype. (f) The expression levels of subtype markers within patients, including ESR1 and PGR for ER+, ERBB2 for HER2+, and VIM for TNBC subtypes. (g) PCA analysis of expression profiles from all patients, colored by subtypes. (h) Percentage distribution of molecular subtypes (LumA, LumB, Her2, Basal, and Normal) assigned to each patient. (i) Spearman correlation coefficient indicating the relationship between CCSs and each molecular subtype.

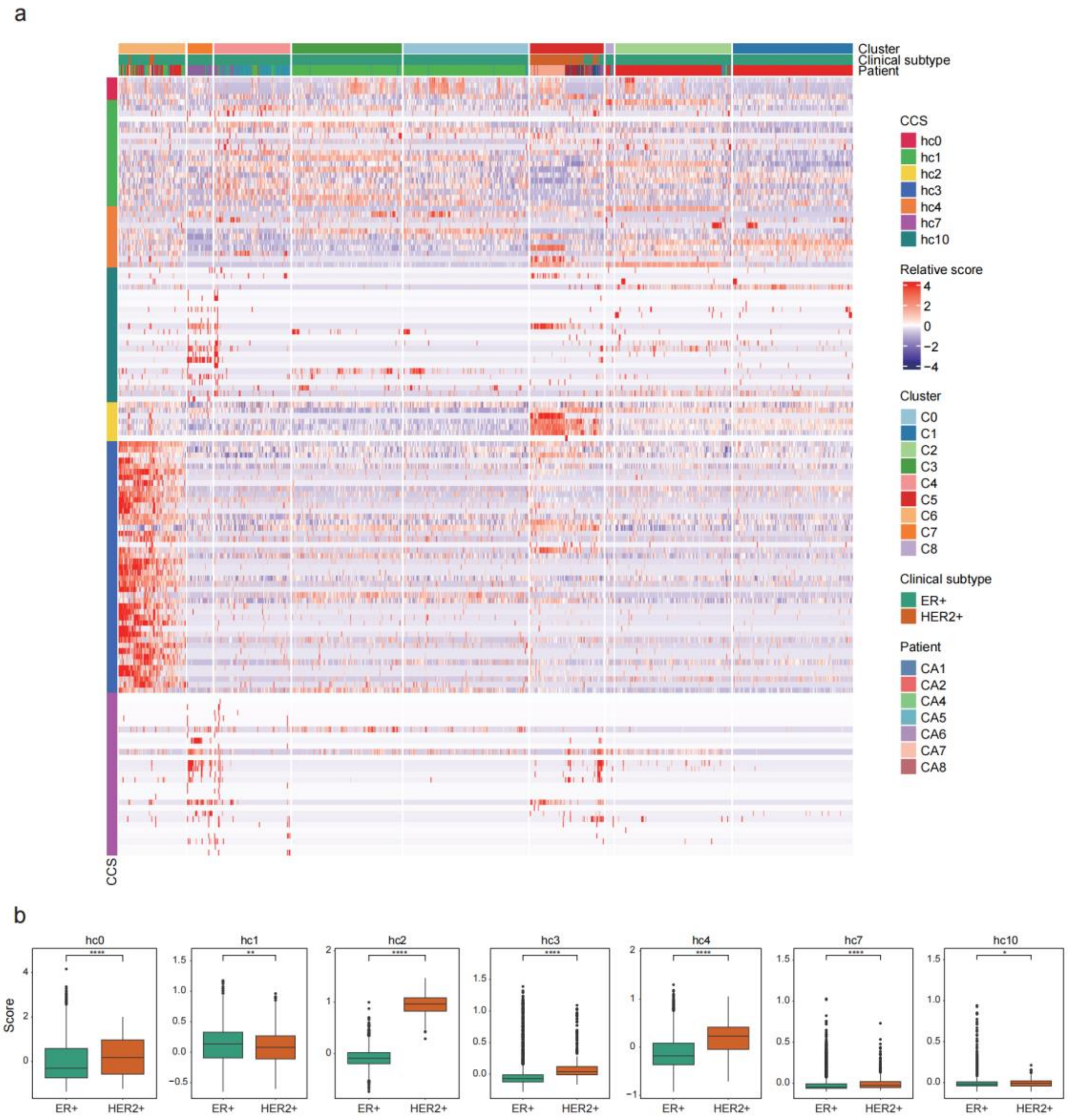

Supplementary Figure 10. Association of CCS with cell clusters and clinical subtypes in the Liu_2022 cohort. (a) The expression levels of signature genes of CCSs within cancer cells of each cluster. (b) Box plots showing expression scores of signature genes of CCSs in the two clinical subtypes (ER+ and HER2+). P values were determined through a two-sided Wilcoxon rank-sum test (*$p < 0.05$, **$p < 0.01$, ***$p < 0.001$, and ****$p < 0.0001$, denoted by asterisks).

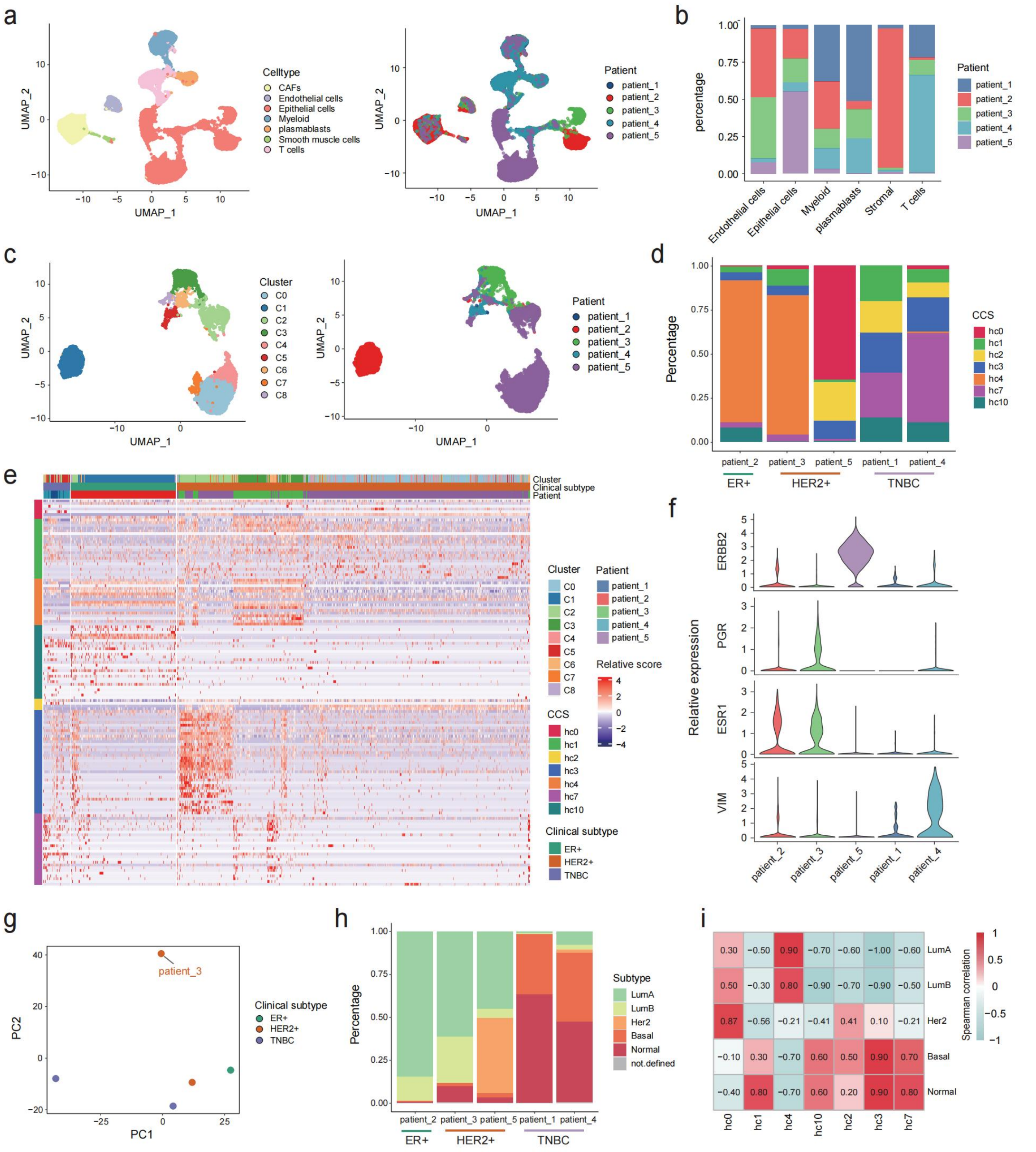

Supplementary Figure 11. The consistency verification of CCSs, clinical subtypes, and molecular subtypes in the Xu_2021 cohort. (a) UMAP visualization of all cells, colored by the major cell types annotated based on canonical markers (left), and patients (right). (b) The percentages of cells from all patients in each major cell type. (c) UMAP visualization of all malignant cells from patients with at least 10 malignant cells, colored by cluster (left) and patient (right). (d) Percentage distribution of CCSs assigned to each patient. (e) The expression levels of signature genes of CCSs within cancer cells of each clinical subtype. (f) The expression levels of subtype markers within patients, including ESR1 and PGR for ER+, ERBB2 for HER2+, and VIM for TNBC subtypes. (g) PCA analysis of expression profiles from all patients, colored by subtypes. (h) Percentage distribution of molecular subtypes (LumA, LumB, Her2, Basal, and Normal) assigned to each tumor. (i) Spearman correlation coefficient indicating the relationship between CCSs and each molecular subtype.

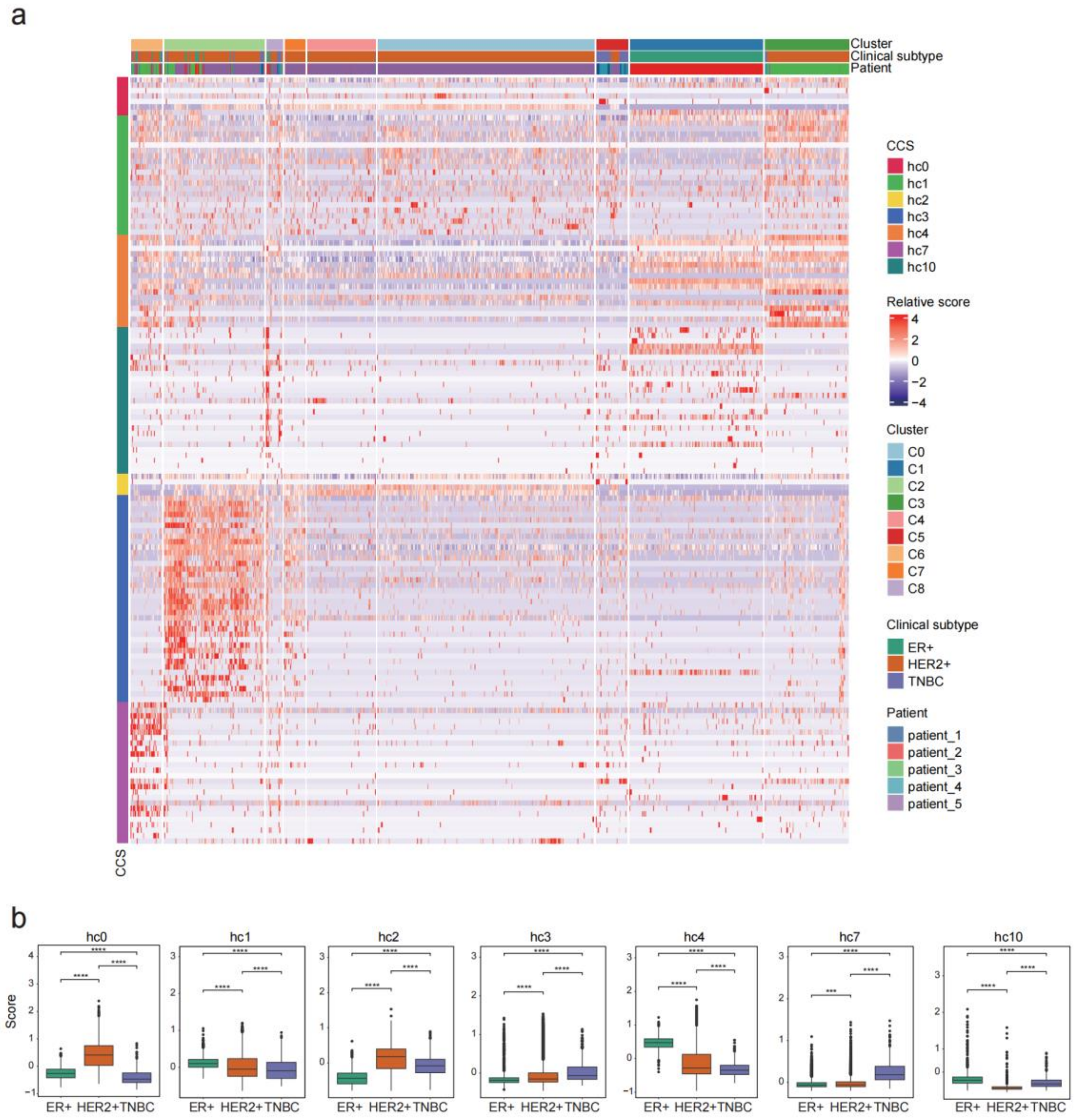

Supplementary Figure 12. Association of CCS with cell clusters and clinical subtypes in the Xu_2021 cohort. (a) The expression levels of signature genes of CCSs within cancer cells of each cluster. (b) Box plots showing expression scores of signature genes of CCSs in the three clinical subtypes (ER+, HER2+ and TNBC). P values were determined through a two-sided Wilcoxon rank-sum test (*p < 0.05, **p < 0.01, ***p < 0.001, and ****p < 0.0001, denoted by asterisks).

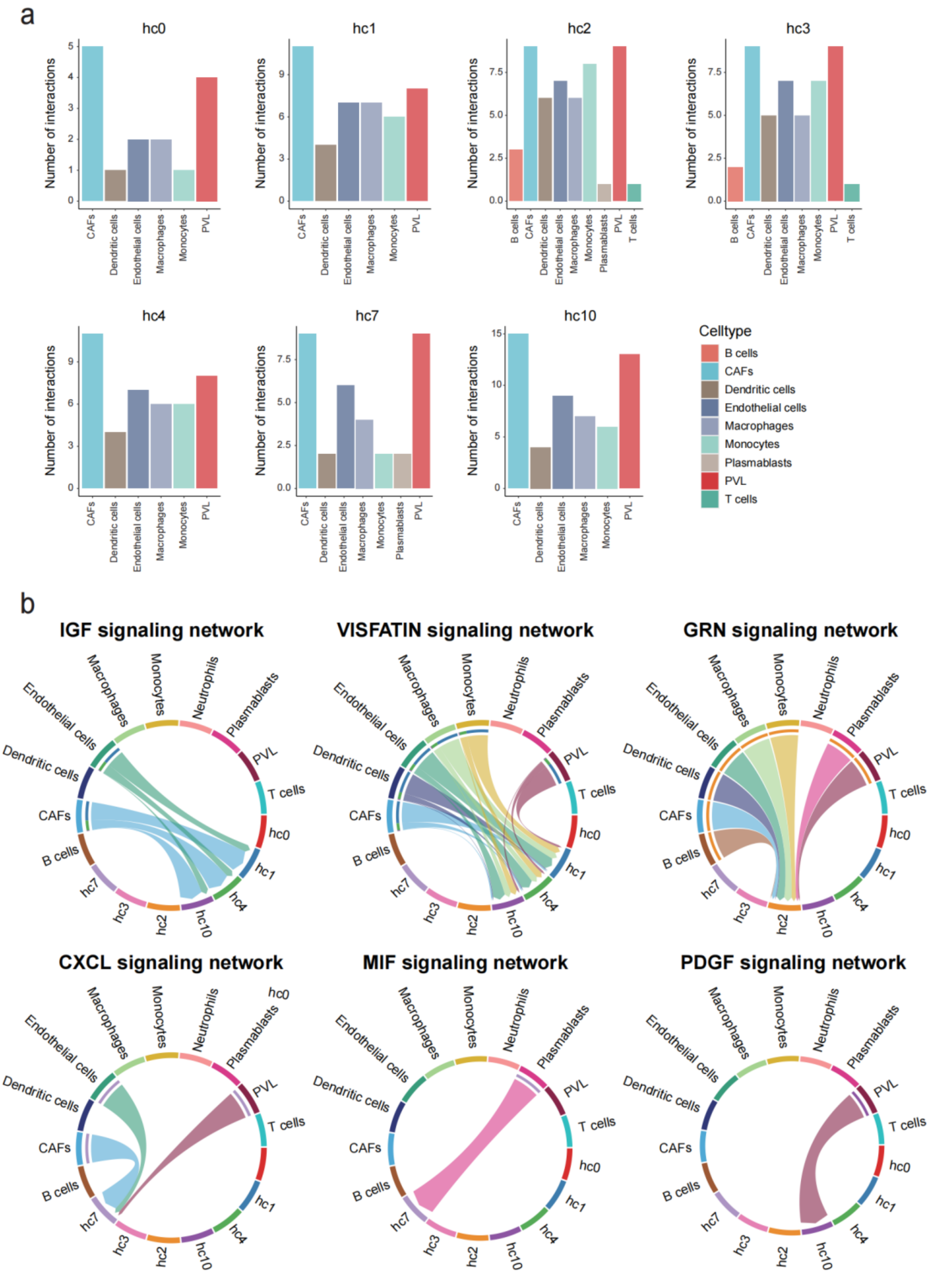

Supplementary Figure 13. The statistical analysis for communications between the non-malignant cell types and CCSs. (a) Bar graphs showing the number of interactions originating from non-malignant cell clusters (B cells, CAFs, dendritic cells, endothelial cells, macrophages, monocytes, plasmablasts, PVL, and T cells) to each CCS. (b) Chord diagrams illustrating the state-specific signaling networks: IGF and VISFATIN for G1 (hc1, hc4 and hc10), GRN for hc2, CXCR and MIF hc7, and PDGF for hc10.

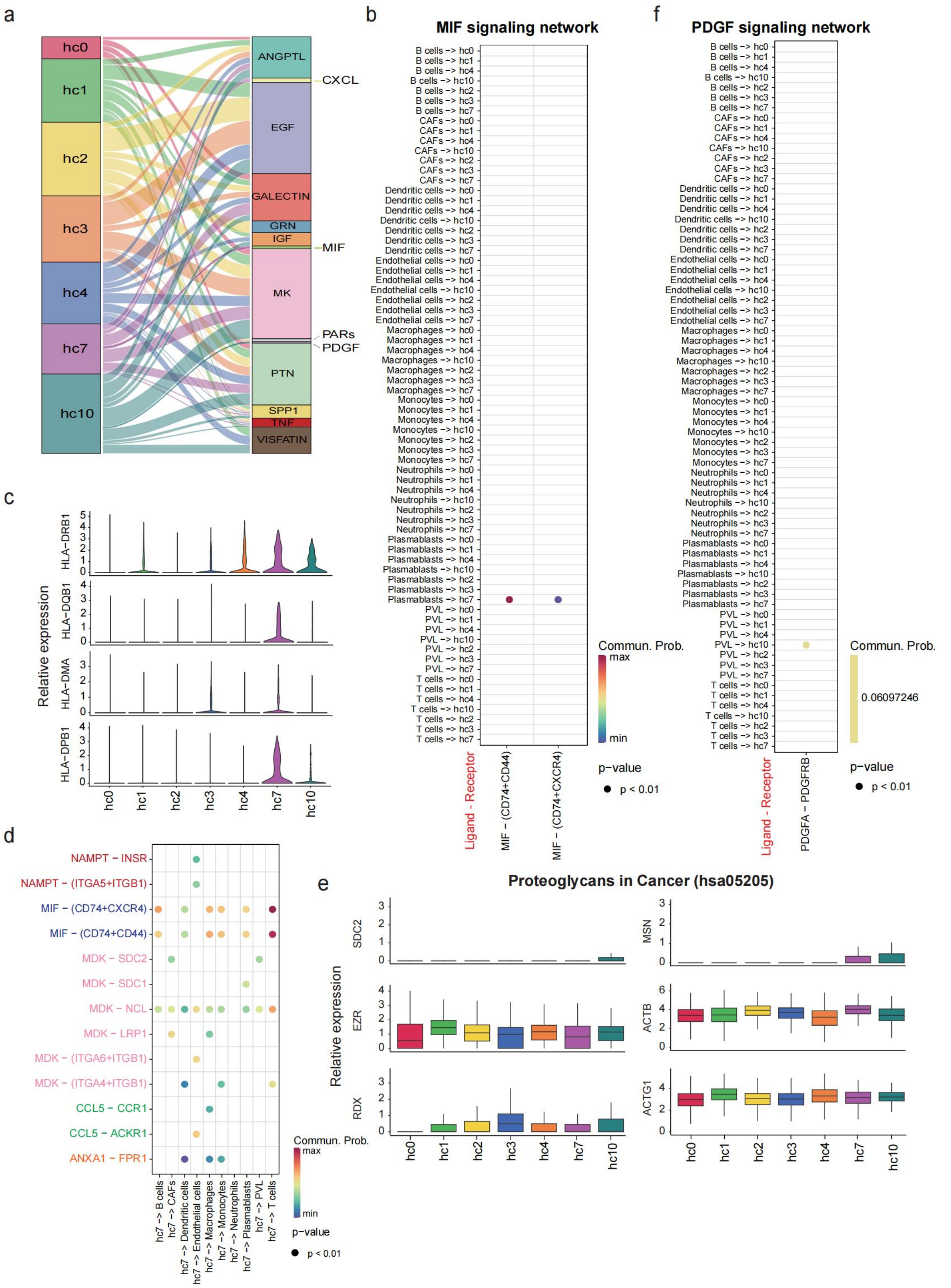

Supplementary Figure 14. Communications from non-malignant cell clusters to hc7 and hc10. (a) Sankey diagrams revealing incoming signaling pathways for each CCS. Specifically, only hc7 was implicated in CXCL and MIF signaling, while hc10 exclusively participated in the PDGF pathway. (b) Plasmablasts engaged with hc7 through specific ligand-receptor pairs, MIF-(CD74+CD44) and MIF-(CD74+CXCR4). (c) Expression of MHCII-related genes across CCSs. (d) Signal identification by comparative analysis of communication probabilities mediated by ligand-receptor pairs between hc7 and non-malignant cell types. (e) The expression levels of SDC2 downstream genes in the KEGG pathway

of proteoglycans in cancer. (f) PVL targeted hc10 exclusively via ligand-receptor pair (PDGFA-PDGFRB) in the PDGF signaling network.

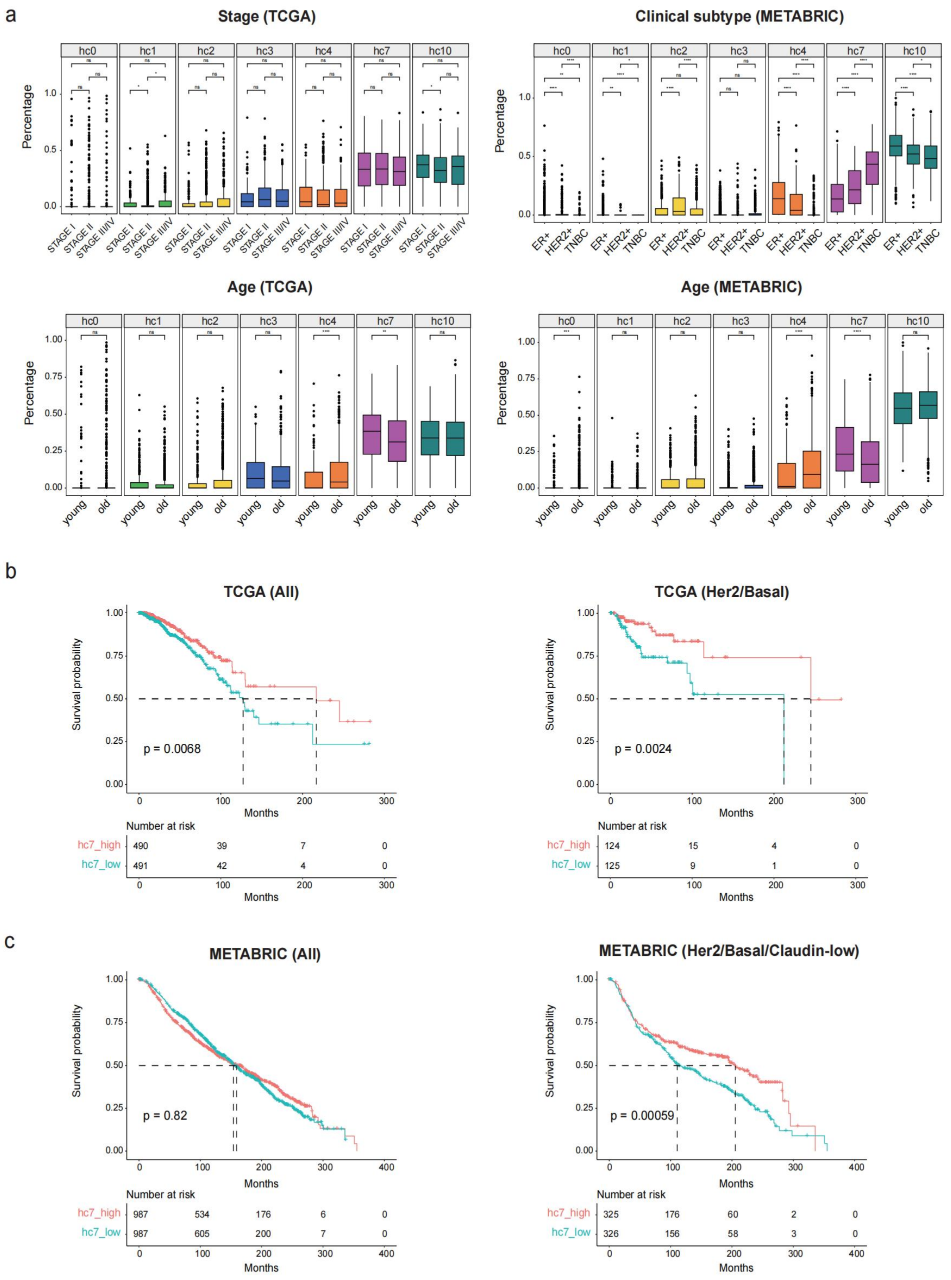

Supplementary Figure 15. Correlations between CCSs and clinical metadata and outcome. (a) Boxplots showing the CCS percentages across different stage groups and age groups in the TCGA cohort (left), and across different clinical subtypes and age groups in the METABRIC cohort (right). P values were calculated by a two-sided Wilcoxon rank-sum test (P-values denoted by asterisks: *$p < 0.05$, **$p < 0.01$, ***$p < 0.001$ and ****$p < 0.0001$.). (b-c) Kaplan-Meier plots illustrating survival outcomes: overall TCGA cohort and TCGA subsets with Her2 or Basal subtypes (b), as well as overall METABRIC cohort and METABRIC subsets with Her2, Basal, or claudin-low subtypes (c). High and low groups were categorized based on the proportion of hc7 inferred in each patient. P-values were determined using the log-rank test.
